# General Index to the British and Foreign Medical Review

**Published:** 1848

**Authors:** 


Dropsy, renal, M. Rayer on . . xv 484
scarlatinal, cause of . . xxiii 424
serous and fibrinous . . xxii 325
succeeding scarlatina . . xiii 261
tincture of broom-seed in . i 534
treatment of . . . viii 319, xxi 370
urine in . . . . xvi 336
uterine, or hydrometra . . xv 105
with renal disease, cases of . ii 226
Drowned, treatment of the . . xxii 165
degree of heat for the . . xxi 271
Drowning, action of the heart in . xxi 269
death by, experiments on . ii 426
fractures in . . . . xxi 485
inflation of oxygen in . . xxi 270
marks on neck in . . . ix 49
morbid appearance from . . iii 564
Mr. Watson on . . . v 176
recovery after . . . xxi 269
sign, as evidence of . . ix 49
Sir B. Brodie on . . . xxii 164
Droste, on softening of mucous membrane . . . . ii 551
Drug diseases, observations on . xxii 455
Drugging, on the atrocities of . xxii 238
Druggists, and apothecaries . . xiii 212
and physicians, connexion of x 220
on the education of . . xxi 265
on the practice of . . . xix 226
Drugs, adulteration of, remarks on . v 161
Arabian, derived from India . xxiii 528
cures ascribed to . . . xxi 518
from animals, prohibited in Russia i 602
interdicted by Priessnitz . xxii 440
on the constant use of . . xxii 170
Druitt, Mr., his memoranda for students . . . . vi 202
his Surgeon's Vademecum viii 536, xii 518
Drummond, Mr., his First Steps to Anatomy . . . . xxi 493
Drum of ear, diagnosis of soundness of vi 143
Drunkards, change of the sensations in ii 479
enlargement of spleen in . . xvi 219
healthy feelings unknown to . ii 480
insensibility to pain in . . ii 480
on asylums for . . . xv 55
Drunkenness, and apoplexy, diagnosis of . . . . xvii 130
habitual, restraint in . . x 136
in relation to malaria . . xviii 505
legal responsibility in x 158-161, 171
mode of curing . . . iii 231
validity of deeds in . . x 158
Drunken person, evidence of a . x 159
Drysdale, Dr., on homœopathy . xxi 225
Dry-cupping, use of, in diseases . xiv 219
Dualistic theory, extension of . xxiv 137
observations on . . xxiv 134
Duality of mind, Dr. Wigan on . xx 1
phenomena like . xx 16
Dublin, a kind of yellow fever in . xvi 240
diseases of, Dr. Rutty on . xviii 189
hospitals, statistics of . . ii 185
Dublin lying-in hospital, bad practice in ii 96-7
economy of, in 1829 ii 94
statistics of . . ii 72
medical degrees, number of, in ten years . . . . i 610
sanitary condition of . . xxi 1, 14
Dubois, M., on etherisation in midwifery . . . . xxiii 569
on medical study . ix 411, x 175
tables of ages of medical men . i 202
Ducamp, M., his filching from Arnott xii 408
his inventions in lithotrity . xii 408
Duchatelet, M., and the Amelot sewer v 450
account of . . . . v 449
on public hygiène . . v 447, xi 1, 13
on the voirie of Montfaucon xi 14, (App.)39
Duck- or splay-foot, treatment of . viii 399
Ducpetiaux, M., his works . . xix 126
on the mortality of Brussels . xix 126
Ducros, M., on turpentine enemata in sciatica . . . . i 569
Duct, common biliary, occlusion of . xxi 54
Ducts, biliary, inflammation of . xxi 52
Ductus arteriosus, anomalies of . viii 22
closure of . . . xvii 274
Ductus choledochus communis, valves in . . . . xii 133
Ductus venosus, closure of . . xvii 273
Duffin, Dr., on strabismus . . xi 474, 492
Duges, Prof., on abortion . . vi 81
Duhamel, on the formation of bone xxiv 398
Dumas and Boussingault, on organic chemistry . . . xviii 531
Dumbness, sudden attack and cessation of . . . . xiv 553
Dumenil, Dr., on chemical tests . vii 533
Dumfries lunatic asylum . . ix 144
Dundee, increase of fever in . . xi 31
lunatic asylum of . . . ix 144, 157
reports of . . . vii 1, 53
three years' fever bill of . . xv 341
Dung-heaps in towns . . xxi 340, 349
Dung, supplies ammonia to plants . xi 441
Dunglison, Dr., his American Library iv 495
his Elements of Hygiène . i 313, 344
Human Physiology . . xviii 530
Medical Lexicon . . viii 548
Student . . iv 496
New Remedies . ix 541 xiii 54, 88
Physiology . v 75, 113, vii 229
Practice of Medicine xiv 220, xviii 285, xx 441
on human health . . . xx 519
on malaria . . . . i 349
on polypus of the heart . . vi 547
on therapeutics, &c. . . xvii 242
Dunlop, Mr. his exertions on temperance . . . . xxiv 521
Dunn, Mr., curious cataleptic case by xxiii 229-34
Duodenitis, a cause of jaundice ix 482, xi 418
a feature of yellow fever . ix 482
Duodenum, colour of, internally . xi 418
in relation to liver disease . xi 418

Duodenum, malformation of, case of xxiii 412

Mr. Mayo on diseases of . iii 74

three portions of . . . xi 418

ulceration of . . . xi 418

in burns . . . . xv 155

DUPARCQUE, M., on diseases of the uterus . . . . ii 511

on rupture of the uterus . x 486, 493

DUPEYRON, M., on sanitary regulations . . . xvi 289, 302

DUPOTET, M., on animal magnetism vii 324, xix 428

DUPUY's experiments on poisons . i 559

DUPUYTREN, fault of, in amputation xiii 173

his new method of lithotomy . ii 99

his staff for lithotomy . . ii 100

on lithotomy instruments . ii 100

the Napoleon of surgery . iii 387

Dura mater, case of fungus of . vi 541

Mr. Guthrie on wounds of xv 177

rheumatism of . . xiv 413

spasm from pinching . xiv 56-8

DURAND-FARDEL, M., on softening of the brain . . . xvi 1

Duration of disease in relation to danger i 408

of life, M. Chadwick on . . xviii 197

probable, in England . xviii 200

Durham cattle, fattening of . . vii 382

DURLACHER, Mr., his treatise on corns xix 560

DÜRR, Dr., on infantile cholera . i 257

Dusty atmosphere, mean age of workmen in . . . . . i 197

Dutch, clothing of the, remarks on i 333

universities . . . . vi 275

Duties and rights of medical men . xxi 438

DUVAL, M., on club-foot, anchylosis, &c. xviii 353

DUVERNEY's glands, description of xvi 156

Dwarf, artificial labour in a . . x 272

Dwarfs and giants, remarks on . viii 18

Dwellings, unwholesome, death from iii 252

Dying, little suffering commonly in viii 274

Dynamic diseases, disbelief in . xxiii 66

school of physiology . . i 384

Dynamics, mental, Mr. Green on . xxiv 216

vital, Unzer's work on . xxiv 181, 183

Dynamico-vegetative pseudoplasm . x 335

Dypsychia, cases of . . . xix 443-5

Dyscrasiæ, &c., report on . . xxiii 308

Dyscrasy, bilious, Dr. Horaczek on xxii 311

case of, treated with sugar . v 243

Dysenteria incruenta, of Willis . xxiv 350

Dysenteric fæces, microscopy of . xxiii 504

ulceration, Dr. Parkes on . xxiii 147

Dysentery, affection of liver in . iii 332

and fever, at Chusan . . xxii 181

and hepatitis of India . . xxiii 146

and tape-worm, bread-pills in xxiii 267

calomel in . . . . xviii 319

case of perforation in . . ix 542

chronic, case of . . . iii 145

ipecacuanha in . . iii 145

mercury in . . . iii 145

treatment of . . . iii 145, 335

Dysentery, combined and complicated xxiv 345

complications and causes of . xxiii 150

compound powder of jalap in . iii 331-3

contagion of, from stools ? . xxiv 357

contagious, fevers with . . xxiv 353

contagiousness of . . . xxiv 352

cures of, without medicine . xxiii 608

dietetic precepts in . . iii 332

diseases of the colon in . . iii 334

Dr. Williams on . . . xiii 98

effect of civilization on . . xviii 253

effervescing draughts in . . iii 183

enema of nitrate of silver in . ii 229

epidemic, in France . . xx 239

four stages of . . . xxiii 148

general remarks on . . xxiv 333

good effect of opium in . . xxi 107

hepatic abscess in . . . xxi 45

pain in relation to . . iii 342

homœopathic treatment of . xxii 579

homœopathy in . . . xxiii 608

in cattle, from bad water . xvii 26

inflammatory nature of . . xviii 317

inflammation of cæcum in . iii 334

intestinal ulcers in . . xviii 317

in the navy, from bad water . xvii 26

Indian and European . . iii 330

in China, morbid anatomy of . xxii 185

in India, treatment of . . xxiii 151

in relation to hepatitis . . iii 334

morbid appearances in . . xviii 316

Mr. Twining, on mercury in . iii 332

on the nature of . . iii 331

treatment of, by . . iii 331

malarious, morbid anatomy of xxiv 349

morbid anatomy of . . xxiv 336

nervous symptoms in . . xxiv 358

nux vomica in . . . i 255

opium in . . . . xviii 318

pathology of . iii 331-2, xxiii 147

putrid, of Bengal . . . xxiv 355

resembling cholera, in 1670 . xxiv 350

scruple doses of calomel in . iii 333

simple, non-contagious . . xxiv 334

the severer forms of . . xxiv 343

treated with albumen . . ix 552

treatises on . . . . xxiv 333

treatment of . . . . xviii 318

use of ipecacuanha in . . iii 331

ulcers in the colon in . . xxiv 336

with hepatic abscess . . xxiv 358

malarious fevers . xxiv 346-52

other abdominal diseases . xxiv 357

typhoid fevers . . xxiv 352

Dysmenorrhea, abortion from . x 595

abortion mistaken for . . vi 84

acute, treatment of . . xviii 524

and rheumatism, connexion of xviii 522

belladonna in . . . x 44

chronic, treatment of . . xviii 524

dilatation in . . . . xxi 89

Dr. Bushnan on . . . x 593

Dr. Rigby on . . . xviii 522

Dysmenorrhea, fibrinous exudations in xviii 523

firm coagula in . . . xi 180

leeches to knee in . . . xiii 236

narcotics in . . . x 595

nervous nature of . . . x 595

observations on . . xiv 388, 390

report on . . . xx 539, xxiii 292

scarifying cervix uteri in . xviii 524

on tinct. guaiac. volat. in . vi 91

treated by bougies . . iii 113

treatment of . . . . viii 40

varieties in . . . . x 594

veratria ointment in . . x 594

Dysmenorrheal membrane . . xi 180

Dyspepsia, a cause of phthisis . i 73

alkaline . . . . ix 273

atonic, dietetic rules in . . xviii 61

caused by irritated brain . iv 62

cause of atrophy in . . ii 463

colonic, the tongue in . . iii 72

cure of, by hydropathy . . xxii 455

Dr. Steward on . . . xxiv 579

from chronic gastritis . . ix 477

disease of par vagum . iii 28

gastritis, remarks on . xii 116

hemorrhoids . . . xix 28

mental causes . . xii 274

mind, treatment of . xii 276

using tobacco . . xix 27

gastric uneasiness in . . xix 28

gastritis in, diagnosis of . xii 116

hygienic cure of . . . vii 478

treatment of . . . xxiii 579

in children, practice in . . xii 165

Sir J. Clark on . . xii 165

investigation of . . . vii 120

lactic acid, a cause of . . xi 343

mental disorder, a cause of . iii 197

on detecting the cause of . vii 120

opposite conditions in . . vii 119

treatment of . . . . ix 477

use of alum in . . . viii 555

mist. ferr. comp. in . xvi 195

nux vomica in . . iv 244

Dyspeptic affections, on alcohol in . xxiv 546

treatment of . . xxiv 546

patients, climates for . . xii 162

phthisis, baths for . . . iv 254

Dyspeptics, constitutions of . . vii 120

Dysphagia in typhoid fever, cause of ii 37

Dyspnea, an occasional cause of . vii 128

from affection of par vagum . iii 27

in first stage of phthisis . . ix 333

in pregnancy, Dr. Meigs on . xxi 90

much relieved by bloodletting i 410

Dystocia, Dr. Naegele on . . xix 60

Dysury, from enlarged prostate . xiv 164

Eagle, Mr., on smallpox . . vii 278

Ear, action of air in the middle . viii 96

anatomy of, history of the . viii 71

and deafness, Mr. Webster on vi 199

Ear, caries in the, cause of . . iii 90

catarrhal inflammation of the . iii 89

cicatrized treatment of the . iii 90

crack in the, advantage of a . vi 200

diagnostic sounds in the, by air viii 99

diseases of . . viii 89, xiii 509, 517

acetate of lead in . . iii 94

ancient authors on . . iii 80

auscultation in . . iii 96

causes of . . . xxiv 35

classification of . . iii 87

curability of . . . iii 85

curable and not . . xxiv 37

Dr. Deleau on . viii 69, 71, 95

duration of . . . xxiv 31

electro-magnetism in . xxiv 40

English authors on . . iii 81

French authors on . . iii 81

in relation to age . . xxiv 30

modern authors on . . iii 80-1

on treating . . . viii 98

origin of all . . . iii 88

prognosis in . . . viii 100

simultaneous . . . xxiv 29

statistics of . . . xxiv 24

treatment of . . . xxiv 36

-doctors, great philanthropy of xiii 500

Dr. Kramer on diseases of the iii 79, v 216

on examining the . . iii 87

inflammations of the . iii 88

noise in the . . iii 98

Dr. Kramer's lamp for examining iii 88

syringe for the . . iii 89

drum of, diagnosis of soundness of vi 143

errors of writers on noise in the iii 98

exploration of the, for diagnosis xxiv 26

external, cartilage of the . viii 73

diseases of the . . iii 87

extracting foreign bodies from the iii 266

functions of semicircular canals of xvi 369

hardened wax in the . . iii 89, 92

injecting etherial vapours into the viii 101-2

internal, artery in the . . viii 77

diagnosis of, diseases of . iii 97

diseases of the . . iii 87, 97

essential part of the . xvi 369

M. Breschet on the . . viii 68

-medicine, contributions to . xxiv 22

Dr. Lincke on . . viii 68, 69

middle, diagnosis in diseases of viii 98-9

diseases of the . . iii 87, 94

inflammations of the . viii 91, 97

movements of bones in the . xiii 526

ossicula of the . . xxii 287

Mr. Cock on malformation of the ii 167

Mr. Pilcher on the . . . viii 68, 70

Mr. Toynbee on pathology of the xviii 370

neglect of due inspection of the iii 87

of fishes, discovered by Hunter xv 213

osseous labyrinth of the . . viii 78

ossicles of the, ligaments of . viii 77

pathological changes in the . xiv 62

periosteal inflammation of the iii 90

Ear, phlegmonous inflammation of the iii 90
physiological considerations on viii 89
physiology of the . . . viii 82
history of the . . viii 82
plastic operations on the . xxi 302
polypi of the . . . . xxiv 38
history of . . iii 89
shape of the, in insanity . xix 596
stomach and lungs, connexion of xvii 414
structure of the, in various animals viii 77
sunlight best for examining the iii 88
-surgeons, English, Kramer's strictures on . . . iii 96, 98
surgery of the, history of . iii 80
syringes for, and syringing the iii 89
table of diseases of the, by Kramer iii 86
lesions of the, causing deafness . . . viii 99
tepid water best injection for the iii 80
treatise on the . . . x 244
true internal inflammation of the iii 97
various treatises on the . . viii 68
use of the external . . viii 83
-wax, use of . . . viii 84
Ears, cough from irritating the . xvii 414
chronic vomiting, from beans in xvii 414
operation for restoring the . vii 411
Eating, best rules for . . . ii 382
education of idiots in . . xxiv 14
rapid, bad effects of . . ii 374
times of . . . . xvii 45
when to desist from . . ii 382
with eighth pair of nerves cut ii 137
Eau de Cologne, stupor from vapour of xviii 564
Eau d'Enghein, its use in lead colic ii 576
Eaux Bonnes, spa at . . . xiv 353
Eble, Dr. on Egyptian ophthalmia x 3, 22
on ophthalmia . . . vi 197
Eberle, his physiology of digestion iv 201
Ebriety in United States' army . xvi 43
Earle, Mr., history and writings of v 627
obituary of . . . . v 627
on contractions from burns . xi 315
Early rising, observations on . . x 46
Earth and sea, temperature of . xxii 229
geological history of the . xix 164
use of, as food . . . xvii 16, 17
Earthy salts of urine, pathology of . xx 67
East Indian command, diseases on the xvii 323
medicine and surgery . xix 72
East Indies, health of navy and army in xv 9
Eccentric persons, validity of wills by x 138
Eccentricities, surgical, M. Mayor's xx 461
Eccentricity and insanity, diagnosis of x 133
Ecchymosis, from ligature on neck . v 176
in infants, from the funis . ii 415
in new-born infants . . vii 90
of the eye, in injuries of the head xii 550
on the neck, in strangulation . ii 419
Echinococci, contained in hydatids . xvii 196
structure of . . . . xvii 198
Echinococcus, account of . . xx 407
-hydatid, researches on . . xvii 194
hominis, Mr. Wilson on . . xxiii 410
Echinococcus, Mr. Owen's account of xvii 195
Echinodermata, osseous tissue of . xxiv 391
Echo, the affection so called . . xvii 422
Eclampsia parturientium . . xvii 422
puerperal, Dr. Meigs on . . xxi 91
Eclectic, and empiric sects, account of xxiii 100
French medical men . . iii 389
of Adone Palmieri, &c. . . i 546
treatment of disease . xv 408, 412
Eclectics, Magendie's tirade against viii 329
Eclipse, solar, effects of, on animals xv 231
Economy of Health, Dr. Johnson's v 380, 393
Ectopia of viscera through diaphragm xxiv 179
Ectromeles, the rare monster . . viii 6
Ectropion cured by autoplasty . xii 252
Ectropium of the lips, operation for xxi 304
operations for . . . x 27
plastic operation for . . xi 75
with eversion, operation for . vii 404
Ecthyma, M. Rayer on . . . ii 153
syphilitica, characters of . xxiii 357
Eczema, acute, of the ears . . xv 116
amiantacea, obstinacy of . xv 117
chronic, of the scalp . . xv 116
furfuracea, obstinacy of . . xv 117
of scalp, diagnosis of . . xv 117
treatment of . . . xv 117
oxide of zinc ointment in . xiii 541
varieties of . . . . xv 115
Ede, Mr., his facts in chemistry . iv 498
Edict, ancient Indian medical . xxiii 522-3
Edinburgh, clinical report on fever iv 247
contagious fever in . . xi 27
distress of lower classes in . xi 25
Dr. Marx's impressions of . xv 19
fetid irrigations around . . xi 1, 19
infirmary, report from . . v 601
lunatic asylum, music in . . xv 282
Medical Dictionary . . i 178
faculty, prizes by the . ii 607
graduations, 1836 . . iii 292
1837 . . iv 563
Society's books, catalogue of iii 476
Pharmacopæia . . . xiii 54, 79
tests in . . . . xiii 80
Royal Medical Society of . iii 582
typhus fever of . . . xii 293
Editions, on pretended new . . xvi 511
on pseudo-second . . . xvii 531
Editor, a prefatory notice by the . xxi 501
correspondence with the, on "Young Physic" . . xxii 243
Editor's acknowledgments to Dr. Marx xv 22
Education, anatomical, remark on . xxi 494
and treatment of idiots . . xxiv 1
at Oxford University . . x 542
best period for . . . ix 412
continental, remarks on . . v 403
Dr. Brigham on . . . iv 55
Dr. Daubeny on . . . x 542
effects of civilization on . xviii 242
from general to special . . x 542
general and special . . ix 413
in America, strictures on . v 413

Education, in England, statistics of . xi 435

influence of, on health . . iv 55

in language . . . ix 415

in number, time, and space . ix 419

intellectual, of the infant . ix 414

in the physical sciences . . ix 420

in physical science . . x 543

in relation to crime . . xxiv 241

medical, addresses on . . xxiii 242

documents on . . viii 287-94

in colleges . . . xxiv 222

Mr. Green on . . . xxiv 216

preliminary . . . ix 411

principles of . . . x 175

remarks on . . . i 2, viii 299

Sir J. Clark on . . xv 530

military medical, in Prussia . i 297

moral and physical . . xv 416

national, Mr. Combe on . . xxiii 254

of children, remarks on . . i 338

idiots, observations on . xix 293

medical men . . . xix 194

new bill on . . xix 549

mothers, Martin on . . xiv 527

physicians and surgeons . xiv 407-8

the intellect . . . ix 412

the observing faculties . ix 414

on phrenological principles . x 243

on preliminary medical . . xx 194

over-, a cause of insanity . iv 61

physical, Dr. Caldwell on . iii 472

of women . . . v 405

remarks on systems of . . iv 62

Rev. Dr. Booth on . . . xxiv 284

self- . . . . . . x 178

systematic, golden rule of . xxiv 218

use of classical . . . x 543

EDMONDS, Mr., on rate of mortality iii 40

EDWARDS, Dr. M., his Elements of Zoology v 204

on heat in animals . . . i 333

EDWARDS on the breath . . i 243

Eel, responding to whistling . . xix 309

spinal cord of . . . v 495

Effluvia, absorbed by the skin . i 331

contagious, dilution of . . xxiii 318

human, fever from (App. to vol.) xi 41

putrid, Dr. S. Smith on . . xi 9-13

effects of . . . xi 9-19

Effusion, serous, in the brain . . iii 310-11

Effusions, in serous cavities, age of . xii 135

Egestion and ingestion, Dr. Hall on xiv 197

Egg, development of, J. Hunter on . xv 216

incubated . . . . ix 396

membranes of, to incised wounds v 576

white of, injected into veins . viii 349

use of, in fractures . . vi 312, 319

Eggs, latent life of . . . v 556

rotten, poisoning by . . ix 267

EGGERT, Dr., obituary and works of v 314

Egotism of many scientific men . xiv 526

puffy, sample of . . . v 389

EGREMONT, Lord, in relation to cowpox vi 487

Egypt, ancient salubrity of . . xxiv 248

Egypt, expedition to, in 1801 . . vi 153

health of Anglo-Indian army in vi 151-4

liberality of the Pasha of . iv 382

mortality in French army in . vi 154

on plague as epidemic in . xxiv 247

plague in, causes of . . xix 366

receptacles for the dead in . xix 48

Egyptian and Negro races, report on xvii 274

ophthalmia, Dr. Eble on . . x 3, 22

prize essay on . . iii 288

origin of the Negro race . . xxiv 444

Egyptians, ancient, observations on xxiv 443

cranium of . . . . xxiv 444

ophthalmia of, account of . xviii 410

EHRENBERG, Dr., on the infusoria . xiv 478

on organic molecules and atoms xi 137

on the brain, &c. . . . vi 393

Eichelkaffee, or acorn coffee . . xvii 378

EISENBERG, Mr., on the human foot xx 521

El Akmar, or red people in Africa . xxiv 447

Elasticity and endosmose . . vii 174

M. Majendie on . . . viii 326

observations on . . . xv 460

Elastic tissue, mode of growth of . ix 515

structure of . . . xiv 273

Elaterium in anasarca, formula of . iii 432

use of its principle . . . x 288

Elbow, case of dislocation of . . ix 556

contracted, myotomy in . . xiii 27

reduction of old luxation of . vi 233

Elbow-joint, contracted, cure of . viii 402

excision of iii 483, vi 581, xii 253, 330

in Glasgow . iii 559

Mr. Fergusson on xv 193

Mr. Liston on . xxii 371

operation for . xx 51

Elder, in scrofula and leucorrhœa . iii 508

juice of the root of, in dropsy vi 221

ELDERTON, Mr., his instrument for stone xii 406

Electrical apparatus for inflammation viii 409

doctrine of Dr. Dickson . . xv 129

lady of Verona . . . viii 407

phenomena of living beings . xxiii 396

properties of the skin . . xx 312

Electric current, interrupted, effects of xxiii 404

physiological action of . xxiii 400

Electric currents in the nerves . xii 245

fishes, Prof. Matteucci on . xxiii 398

Electricity, and caloric, identity of . xviii 518

and chemical affinity . . xi 139

magnetism, connexion of . xvii 448

religion, Dr. Dickson on . xv 129

animal, Dr. Coudret on . . viii 406

as a remedial agent . . xix 368

effects of, on nerves . . xix 575

on absorption . . viii 321

evolution of, by the body . xiv 562

for deafness, inefficacy of . iii 84

from friction of animals . . viii 407

hastens putrefaction . . ii 397

human, remarkable case of . vi 249

induction of, by heat . . viii 411

inflammation caused by . . viii 408

Electricity, in relation to diseases . viii 440
in typhus fever . . . viii 445
in relation to epidemics . . vii 113
its influence as a remedy . . vi 72
on religion . . xv 129
muscular current of . . xxiii 396
of the torpedo . . . xii 129, 132
physiological effects of . . viii 412
present condition of . . v 323
the motive agent of nerves . viii 406
therapeutical effects of . . viii 412
therapeutic use of . . . xxiii 405
voltaic, Dr. Philip on . . ii 147
use of, as a remedy . . xii 344
Electro-magnetism, at Meath hospital xvi 257
in ear-diseases . . xxiv 40
Electro-metallurgy, Mr. Smee's . xv 541
treatise on . . . xi 510
Electro-physiology of man . . xvii 246
Electrometer, medical, of Dr. Coudret viii 409
Electroscope, from frog's leg . . xxiii 396
Electrotype, observations on . . xi 510
Electrotyped copper utensils, danger of xviii 552
Elementary constituents of body xix 249, xxi 541
forms of disease, Dr. Carswell on i 117
Elements discharged in excretions . xxii 262
of diseases, primary . . xvii 474-5
proximate . xvii 474-5, 486
secondary . xvii 474-5, 486
structural . . xvii 498
of medicine, by Dr. Bene . i 1
Elephant, example of a reasoning . xi 93
Elephant's flesh, as food . . xvii 16
Elephantiasis, anaisthetos, seu glabra xviii 347
arsenic and mudàrr in . . xii 425
at Patna, in India . . . xix 73
case of, in Berlin . . . xxiv 48
causes of . . . . . xii 425
cure of . . . . . . xii 425
Græcorum . . . . . xx 267
Dr. Boeck on . . . xviii 346
in China, account of . . xxii 187
in France and Italy . . xviii 352
non-contagiousness of . . xii 425
of labia pudendi . . . xx 548
remarkable case of . . . vii 578
symptoms of . . . . . xii 424
Elixir, Haller's acid . . . vi 329
Elkington, Mr., on the skeleton . iii 205
Elliot, Mr., on squinting . . xi 474
Elliotson, Dr., and animal magnetism . . . . vii 348, 351
his egotism . . . . . xi 498
his lectures . . . . ix 461, 494
his physiology . . . xi 497
on creosote . . . . ii 168
on mesmeric diagnosis . . xx 287
Ellis, Mr., his anatomy . . xi 220
on clinical surgery . . . xxiii 250
Ellis, Mrs., on the South of France xiv 354
Ellis, Sir W., on insanity . . vii 1, 47
on lunatic asylums . . ix 144, 180
Elm-bark, bougies, &c. made of . vi 259
Elsaesser, Dr., on soft occiput . xvii 371
Elytorrhaphie for prolapsus uteri . xxi 326
Emaciation in phthisis, opposition to xxii 88
Emaillottage, hydropathic . . xii 191
Emanations, putrescent, danger from xxi 335
Embalmed bodies, examination of . v 221
Embrocation for sero-cystic tumours xxii 167
Embryogeny, Atlas of . . . ix 2
M. Coste on . . . . ix 2
Embryo, and ovum, physiology of . xix 587
development of . . . xv 266
early development of . . i 238
first appearance of . . . ix 4
food and excretions of . . xix 592
form of stomach of human . ii 541
human, encephalon of . xxii 497, 502
in the ovum, formation of . ix 508
M. Baer on heart of . . i 241
respiration of . . . xix 592
sex of, Dr. Carus on . . i 101
vesicula umbilicalis in the . i 239
Embryology, Dr. Barry on . . ix 2
founded by Aristotle . . i 380
researches in . . . ix 291
Embryonic development of human brain . . . . . xxii 502
structures, on nutrition in . xx 125
Embryotomy, remarkable case of . vi 237
Embryulcia, Dr. Collins on . . ii 80
Prof. Kilian on . . . iii 421
Emesis by tobacco on epigastrium . xv 73
Emetic of salt and water . . v 390
Emetics, administration of . . xv 73
in clyster, uncertain . . xv 73
inefficacy of, in some stomachs ii 542
means of aiding . . . xv 73
operation of . ii 542
medicinal . . . . . xv 72
opium the night before . . xv 73
use and abuse of . . . xv 71
of in bronchitis . . xx 245
phthisis . . . xviii 160
typhus fever . . viii 438
Emetic tartar, action of, in pneumonia xx 81
and gastric irritation . i 423
henbane, Dr. Philip on ii 146
opium in fever . xvi 238
opium, use of . v 275
discordant opinions on . i 417
how beneficial . . i 423
in affections of head . ii 146
bronchitis . . i 421
cerebral inflammation i 421
delirium tremens . i 421
erysipelas of eyelids . x 25
inflamed mamma . iv 533
pneumonia, M. Louis on i 414
puerperal convulsions . ii 87
in synovial effusions . vi 224
large doses of, by Rasori i 419
mechanical action of . viii 251
Odier's mode of exhibiting iii 458
ointment, in uterine disease ii 517

Emetic tartar, ointment, Jenner's . iv 119

strength of . . iv 119

opium with large doses of i 420

peculiar use of . . vii 125

poisoning by . . xviii 552

preparation of . . xiii 60

Rasori's practice with . i 418

tolerance of, remarks on . i 422

vehicles for . . . i 420

with and without bleeding i 421

EMILIANI, Prof., his medical history xxiii 86, 89

Emmenagogue, a remarkable one . vii 234

Emmenagogues, in chlorosis, fit time for xi 178

observations on . . . xvi 193

Emmenological questions . . vii 233

EMMERT, Dr., on pathology, &c. . xvi 267

EMMERT, M., on the nerves . . vi 394

EMMERT's experiments on poisons i 559

Emolument, on professional . . xvii 192

Emotional and instinctive acts . xix 306

Emotional functions, Dr. Hall on . xvi 165

Emotion, expression of . . xviii 449

Emotions, and passions, on the . v 504

Unzer on the . xxiv 200

and propensities, nature of . xxii 513

movements from . . . ix 103

nervous effects of . . . xi 456

relief of, by venting them . xxii 516

true, on formation of . . xxiii 235

EMPEDOCLES, suicide of . . xii 149

Emphysema, abdominal, case of . xxi 470

and phthisis, diagnosis of . xx 448

Dr. Walshe on . . . xviii 164

forced expirations in . . iv 313

friction-sound in . . . xix 113

from deformity of chest . xvii 296

from wounds of lungs . . xxii 323

in lungs, cause of . . . i 482

in typhoid fever . . . ii 51

Mr. Sibson on . . . xix 107

of lung, pathognomonic sign of vii 365

of lungs, anatomy of . . xv 87

causes of . . . xv 87

effects of on chest . . vii 364

hereditary . . . xi 450

M. Louis on . . . vi 28

remarks on . . i 6, 22, xi 448

with tubercle . . xi 410

physical signs of . . xix 108

position in dyspnœa from . xix 108

pulmonary, anatomy of . vii 517

appendices in . . vi 30

atrophy in . . . vii 518

bleeding in . . . vi 40

causes of . . vi 39, xi 406

cough in . . . vi 37

danger in . . . xviii 163

diagnosis in . . . vi 38

Dr. Stokes on . . iv 312

Dr. Lombard on . . vii 516

dyspnœa in . . . vi 32

errors respecting . . vii 519

extent of . . . vi 30

Emphysema, pulmonary, form of thorax in vi 33

frequency of . . vi 40

hereditary . . . vi 40

history of . . . vi 29

hypertrophy in . . vii 519

M. Andral on . . iv 313

M. Louis on . . iv 313

nature of . . . vii 518

pain in . . . vi 37

percussion in . . vi 35

physical signs in . . vi 35-7

progress of . . . vii 518

queries on . . . xviii 162

site of air in . . . xviii 163

sputa in . . . vi 37

sudden death in . . xviii 163

the bronchi in . . vi 31

the heart in . . . vi 32, 38

three forms of . . vii 516

treatment of . . vi 40

tubercles in . . . vi 31, 39

vesicles empty in . . vi 40

use of opium in . . vi 40

subcutaneous, case of . . xiii 506

with meningitis cerebralis . xxii 460

Emphysematous lungs, in infanticide iv 91

Empirical art, characteristics of . xxii 240

credulity in Britain . . ix 465

laws, observations on . . xx 142

pathology, observations on . xxiv 294

practice, hypothesis in . . xx 144

treatment, advocated by M. Louis i 402

remarks on . . . xxi 519

Empiric and eclectic sects, account of xxiii 100

Empiricism, M. Renouard's remarks on xxiii 101

observations on . . . xiv 140

rational . . . . x 183

remarks on . . . xiii 472

Empirics, of the present day . . vi 111

on the success of . . xiv 422, 426

Emplastrum cantharidis, Dr. Spillan on iv 113

lithargyri, improved . . xiii 84

Emplâtre de Vigo in smallpox . xi 246

Employments, effect of, on health . xx 421

Empyema, and pneumothorax, case of ix 394

case of . . . . . vi 266

diagnosis of . . . . xx 248

digitalis and iodine in . . xiii 367

Dr. Hodgkin on . . . iv 44

Dr. Hope's success in . . xiii 366

Dr. Walshe on treatment of . xiii 367

evacuation in . . . . xx 249

injections in . . . xiii 372

new mode of treating . v 242, 573

new operation for . . xii 251

observations on . . . x 287

on the use of the term . . xiii 364

operation for, Mr. Liston on . v 468

paracentesis in . . . xx 450

partial evacuation in . . xiii 370

position of the heart in . . xiii 366

sounds in the stages of . xiii 365

successful treatment of . xiii 366, xxiv 74

Empyema, three sub-stages of . xiii 365

treatises on . . . . . xiii 364

vented by mouth, cases of . iv 45

use of blisters in . . . xiii 368

utility of paracentesis in . xiii 369

Ems, mineral springs of . . xiv 350

waters of, Dr. Granville on . xiv 351

Emulgent veins, diseases of the . xv 486

Enamel of teeth, production of . xx 389

structure of . . . . xx 381

Encephalic bellows-sound . . i 485

congestions, in anemia . . xvii 488

phrenology is in its infancy . xxii 544

diseases, incisions of scalp in . xxi 273

Encephalitis, acute . . . xx 19

Dr. Neisser on . . . xxii 407

in children . . . xx 554, xxi 385

paralytic . . . . xx 30, 37

special diagnosis of . . vi 144

subacute, case of . . . xxii 400

Encephaloid cancer, anatomy of . xxi 423

tumour, constituents of . xvii 446

Encephalomacia . . . . xi 464

senilis . . . . . . xvii 112

Encephalon, cancer of . . . xxii 309

component parts of . . xxiv 577

error in the word . . . xii 100

functions of the . . . x 554

of carp, interesting . . xxiv 574

of fishes, observations on . xxii 495

study of, use of comparative anatomy in . . . xxii 489

vivisections of the . . . xxii 493

Encephalopathia, comatose . . x 456

delirious form of . . . x 456

from lead . . . . x 454

[illegible]gnosis of . . . x 458

duration of . . . x 458

post-mortem appearances in x 459

prognosis of . . . x 458

symptoms of . . . x 457

treatment of . . . x 460

Encephalum, the source of vital power i 558

Encyclopædia of state medicine . xv 225

Encysted tumours, on ossification of xviii 369

Endemic agencies, effect of civilization on . . . . xviii 244

Endemics, epidemic aggravations of xv 5

Endermic absorption . . . vi 340

applications, local effects of . v 345

symptoms from . . xiv 531

inspersion, best time for . v 347

method, absorption in the . v 346

disadvantages of the . v 353

dissertation on the . . v 343

dressings in the . . v 345

first step in . . . v 344

first use of the . . v 343

main advantages of . . v 347

mode of using the . . v 345

Schubert on the . . xiv 530

tepid bath in the . . v 347

with gases . . . v 345

Endermic remedies, action of . v 346

use, best medicines for . . v 344

of various medicines . v 348-52

Endo- and exo-skeletons of animals xxiii 474

Endocardial, and exocardial, sounds xx 178

murmur in rheumatism . . xx 181

Endocarditis, and pericarditis . xx 188

bellows-sound in . . . ii 327-28

case of . . . . . xix 367

causes of . . . . ii 329

complicated case of . . ii 322

confined to right side . . xx 223

deposits from . . . xxiii 170

effects of . . . . xxiii 174

in children . . . . xvii 560

incomplete cures in . . xxiii 169

in pneumonia, Dr. Taylor on . xxiv 562

M. Bouillaud on . . i 426, ii 321

nature and signs of . . xx 457

old and recent, sounds in . xxiii 173

state of the blood in . . xvii 433

symptoms of acute . . ii 326

symptoms of subacute, &c. . ii 327

treatment of . . . . ii 329

vegetations from . . . ii 325

use of mercury in . . . xx 191

with adhesion of valves . . ii 323

Endocardium, earthy concretions of xxiv 120

of the heart . . . . i 431

permanent unsoundness of . xxiii 175

plastic exudations of . . xiv 119

redness of the . . . ii 324

structure and diseases of the . i 431

Endolymph of internal ear . . viii 79

Endometritis, Dr. Kiwisch on . xiii 121

extending to vagina . . xiii 122

Endo-pericarditis, Dr. Hope on . viii 462

physical signs of . . . viii 463

Endo-skeleton, mammal, segment of, *fig.* of . . . . xxiii 480

Endosmose, certain affinities from . vii 174

in membranes . . xxiii 387

in relation to absorption . . xxiii 390

in relation to purgatives . xxiii 390

phenomena of . . . xxiii 385

Endosmosis, and exosmosis . . viii 317

of the egg, experiments on . ix 290

Endosmotic power of various solutions . . . . xxiii 386

Endurcissement lardaciforme, of infants' lungs . . . ii 410

Enema, at commencement of labour xix 71

of tobacco, poisoning from . xii 562

Enema tabaci, properties and use of iv 114

Enemata, mode of using . . v 392

turpentine, in sciatica . . i 569

Energy, objection to the term . iii 22

Enfans, l'Hôpital des, account of . i 231

Engel, Dr., on fatty muscle . . xiv 543

Engelhart, his prediction in 1656 i 601

Engelhardt, M., on spinal cord . xii 235

Engelman, Dr., on the baths of Creuznach . . xiv 310, 320

Enghien, Duke d', case of his playmate vii 46

England, Dr. Marx's recollections of xv 19

vital statistics of . . . xviii 197

English and Americans, activity of xiv 310

English biographies, puerilities in . iv 76

cities, mortality in six . . iii 50

medical practice, Dr. Marx on xv 29

physicians, Dr. Marx's opinion of xv 27

practice, besetting sins of . xxi 263

Engorgement of the lungs . . xi 406

Ensiform cartilage causing pyrosis xiii 558

Enteric disorder, causing insanity . vii 29

Enteritis, case of . . . . xxi 98

case with symptoms of . . xxi 94

compared with typhoid fever . xii 43-44

Dr. Puchelt's divisions of . xviii 311

follicular, disquisition on . ii 69

in children . . . xvii 560

mischief from cathartics in . viii 497

treatment of . . xviii 314, xxi 93

Enterocele, vaginal, in labour . xxi 87

Entero-colitis of infants . . xx 360

treatment of . xx 361

Enterodynia, case of . . . viii 358

hydrocyanic acid in . . viii 358

Enterorrhagy, M. Piorry on . . vi 139

Enterotome, caution in the use of . xv 40

Entero-vaginal hernia, case of . iii 396

Dr. Davis on . iii 396-7

Entomological Society, Transactions of the . . . . . xii 219

Entophytes of vegetables . . iv 19, 20

Entozoa, development of . . xiv 547

frequency of, in man . . ix 442

human, Klencke on . . xx 406

in blood of fishes, and water . xx 416

in the eye . . . . . xviii 422

in the kidneys . . . xv 491

perforations by . . . vii 224

sarconotic . . . . . xxiii 58

seminal, remarks on . . viii 141

tubercle and cancer classed as . ii 598

Entozoon in the eye of a horse . xiii 526

in urethra of a girl . . ix 442

Entropium, Mr. Tyrrell on . . xi 75

operation for . . x 28, xi 75

Enuresis in children, report on . xx 559

case of, by Dr. Roots . . iii 150

nocturna in children . . xvii 561

Epencephalic arch in the cod, *fig.* of xxiii 485

Eperon, the, in artificial anus, Mr.

Trant's instrument for . xxiii 27

Epidemic at Teheran, Dr. Bell on an xviii 369

constitution of 1802 . . i 250

of Berlin, 1837 . . vi 220

of the year . . . . xx 316

on animals . . . vi 220

diseases, characters of . . xxiv 251

essays on . . . xix 365

observations on . . xxiii 314

epistaxis, in Italy . . . xxi 367

in 1494, not syphilis . . xxiii 347

meningitis, in France . . xxiii 82

Epidemics, bygone, relics of . . v 193

in relation to electricity . vii 113

to geology . . . xiii 499

of the middle ages . . . xvii 522

of York, in former times . xviii 503

on periodic returns of . . xviii 172

prevention of . . . xvi 207

researches on . . . xi 515

volcanic origin of . . . xiii 497

Epidermic tumours, Dr. Lebert on . xxii 30

Epidermis, anatomy of the . . ii 446

an organic substance . . ii 453

an organized substance . . vi 334

development of . . . xxi 547

discordant opinions on the . ii 429

Dr. A. Combe on the . . i 329

exfoliation of, in infants . xi 200

external and internal . . vii 239

formation of new . . . xviii 273

intimate structure of . . vi 333

nucleated cells of the . . ix 510

of the negro . . . ii 448

or cuticle . . . . xv 379

permeability of, by fluids . vi 334

production of the . . . ii 446

structure of, in man . . ii 448

in the whale . . . ii 447

vascularity of the . . . vi 333

wanting, in a newborn child . xxiii 298

Epidydimis, encysted hydrocele of . xvii 72

Epidymitis, blenorrhagic . . xii 371

Ricord on . . . x 392

Epiglottis, acute inflammation of . vi 261

elastic polypus from . . iii 72

on the functions of the . . xiii 227

Epilepsy, and hysteria, diagnosis of xvii 134

and mania, digitalis in . . xx 261

Baron Sloet's cure for . . iii 263

caution on ligature in . . xvii 423

closure of the glottis in . . xvi 170

curious case of . . . xxi 286

Dr. Babington on . . . xii 340

epidemic, in schools . . vi 531

fit of, not to be curbed . . xvii 423

from drinking spirits, cure of . xxi 417

from gastric irritation . . xix 32

lead poisoning . . x 457

undue lactation . . xii 329

generally ends in insanity . x 171

hereditary nature of . . xii 104

influence of sex on . . xii 71

interesting case of . . . xvii 423

intermittent character of . xii 341

ligature of arteries in . . xii 556

manifold remedies for . . xvii 423

M. S. Pinel on . . . xx 31

nature of . . . . xii 341

and causes of . . . xv 455

periodic phenomena in . . xxiv 42

remarkable case of . . . xii 341

report on . . . . xx 261

sincipital combustion in . . vii 63

sulphate of zinc in . . . xii 342

Epilepsy, treated with belladonna . ix 252
treatment of . . . xii 341, xx 38
use of digitalis in . . . xi 509
indigo in . . . ii 244
Epileptic convulsions from exostosis xvii 203
fits, palpitation before . . xvii 497
lunatics, observations on . . xix 288
Epileptics, insane, management of . ix 157
Epilogism of the empirics . . xxiii 101
Epiphora, Mr. Tyrrell on . . xi 77
Epiphyses of bones, separation of . v 250
Epiphysis of os femoris, separation of viii 279
Epiphytes, Prof. Vogel on . . xxii 336
Epps, Dr., on counter-irritation . vii 56, 72
Episioraphy, case of delivery after . iii 524
Dr. Fricke on . . . . vii 562
Episiorrhaphie for prolapsus uteri . xxi 327
Epispadias, operation for . . xxi 314
Epispastic liniment of ipecacuanha . xvii 554
Epistaxis, curious causes of . . xxi 367
great losses of blood by . . xxi 368
intermittent, cured by quinine . iii 511
new mode of stopping . . iv 231
plug of amadou in . . . xxiii 375
simple means of arresting . xiv 550
treatment of . . . . xxi 367
Episternal bones, two new . . viii 248
Epithelia, ciliary, in nasal catarrh . xvii 520
morbid, in the sputa . . xvii 519
Epithelial cells, development of . xv 278
tumours, Dr. Lebert on . . xxii 30
Epithelium, ciliary, structure of . xiv 264
cylinder, structure of . . xiv 264
formation of new . . . xviii 273
Mr. Nasmyth on . . . xii 493
nucleated cells of . . . ix 510
tesselated, or plaster . . xiv 264
Epizootic aphtha, in cows, account of xiv 400
disease in Bengal . . . vi 486
in India . . . xx 352
Epizootics, intermittence in . . iv 163
Epulis, cancerous . . . . xxii 294
Dr. Macfarlane on . . . iii 559
Equarrisseurs of Montfaucon, health of xi 15
Equatorial zone, animals of . . iii 372
Equilibrium, and repose, in nature . xxi 117
preservation of . . . v 518
Equitable Society, assurance tables of iii 40
Equivocal generation, remarks on . i 392
Erectile hemorrhoidal tumours, case of xxii 474
tissue, structure of . . . xiv 294
tumour, case of . . . xix 540
in popliteal space . . xviii 367
of uterus, hemorrhage from ii 571
tumours, Abernethy's treatment of ii 565
cure of . . . . ii 564
Dr. M. Hall on cure of . ii 565
of breast . . . xxii 157
Prof. Chelius on . . ii 260
Erect position of body explained . v 135
Eremacausis, process of . . xi 442-3
Erethism, state of . . . . xxiv 301
Erethismus, mercurial, remarks on . v 23
Ergot of rye, antidotes of . . x 555
child's death from . . iii 165
dangerous use of . . iii 164
death of a child from . xiii 71
decomposition of . . xiii 71
difference in strength of . iv 99
Dr. Davis's sweeping use of iii 407
Dr. Hamilton on . . iv 402
Dr. Ramsbotham on . xv 527
Dr. Ryan's doses of . ii 525
effects of . . . xiii 570
essay on . . . xii 509
exhibition of, in clyster . xiv 370
experiments on . . x 554
fed on by an acarus . xiii 70
gangrene from, query on . xvii 501
in menorrhagia . . x 44
in polypus uteri . . iv 370
in post-partum hemorrhage xxiii 289
lessens the heart's action ii 84
numbers of still-born from iii 165
points in the use of . xiii 72
poisoning of fœtus by . xx 528
production of . . xiii 255
remarks on use of . . xxii 54
sedative effects of . . ix 563
successful use of . . v 577
use of, in abortion . . vi 86
in inducing abortion . ii 276
labour . . . xii 479
menorrhagia . iv 369
retained placenta . ii 524
uterine hemorrhage xix 421
watery extract of . . xvii 545
wine of, formula for . xiv 440
with borax . . . vi 86
Ergotine, use of, in menorrhagia . xxiii 292
Erichsen, Mr., on aneurism . . xx 206
on asphyxia . . . xxi 265, 555
congestive pneumonia . xviii 365
diseases of scalp . xv 114, 123
Erigeron acre, noxious to wheat . iv 130
Erotic longings of the aged . . xvii 84
Erotomania, Dr. Davis on . . iii 403
Error, formula for the limits of . xii 20
Erskine, Dr., vid. Areskin . . i 604
Eructations, tremendous, case of . xii 565
Eruption, peculiar, in typhoid fever ii 36, 40
Eruptions, chronic, safety of curing vii 58
on children's heads, remark on xxiii 509
pustular, artificial . . . xv 390
relative prevalence of . . vi 27
syphilitic, Cazenave on . . xxiii 345
treatment in . . . v 25
Eruptive diseases, Liebig's theory on xv 381
fevers, Dr. Chapman's lectures on xxi 358
fevers, see Fevers, eruptive.
Erysipelas, absence of barrier-line in ii 24
and phlegmon, distinction of . xi 326
a nervous inflammation (?) . ii 25-6
antiphlogistic treatment of . xi 325
bleeding in . . . . xi 326-7
bloodletting in . . . v 366-7

Erysipelas, caused by a morbid poison . ii 487
causes of . . . . xi 322
cold affusion in . . . iii 117
and tepid lotions in . xiii 381
conflicting opinions on . . xiii 373
contagion in . . . . . xiii 375
contagiousness of . . . xi 323
contagious property of . . ii 24
cured by electricity . . viii 409
danger of spirituous lotions in . xi 327
definitions of . . . . xi 322
diseases, species of . . xiii 374
disputes on treatment of . . xi 325
divisions of . . . . . xi 322
Dr. Donellan on . . xiii 373, 382
Dr. Mackintosh on . . iii 109
Dr. Williams on . iii 147-8, xi 325
erratic, march of . . . xii 68
most dangerous . . ii 23
facial, a fatal sign in typhoid fever ii 39
sinapisms to feet in . . ii 61
free incisions in . . . xi 328
frequently contagious . . i 373
gangrenous, diagnosis of . ii 23
general treatment of . . xiii 376
hypercatharsis often fatal in . ii 26
idiopathic, causes of . . ii 25
incisions in . . . . . xiii 380
Mr. Travers on . . ii 27
proper extent of . . ii 27
indications for cure of . . xiii 377
infantine, report on . . xvii 550
infectious nature of . . v 365
in infants, fatality of . . xiii 376
remarks on . . . xxii 548
in relation to primæ viæ . . vi 143
its connexion with puerperal fever ii 487
leeches and punctures in . xiii 380
local differences of . . . iii 459
treatment of . . . xiii 380
loss of blood often fatal in . ii 26-7
mercurials in . . . xiii 379
metastasis in . . . xiii 377
mode of using caustic in . . v 608
Mr. Nunneley on . . . xiii 373
Mr. Travers on . . . ii 23
neonatorum . . . . xxi 467
nitrate of silver in . . . xi 328
objection to bark in . . xi 326
obscure pathology of . . ii 25
observations on . . . xi 221
of eyelids . . . . . x 25
treatment of . . . x 25
of nose, from anger . . xi 323
of reflected membranes . . ii 24
of skin and cellular membrane ii 23
on arresting its progress . xiii 377
on the lancet or knife in . xi 328
opium with ammonia in . xiii 380
parts disposed to . . . ii 23
pathology of, Mr. Travers on . ii 25
protoxide of iron in . . xiv 232
puerperal . . . . xiii 120
Erysipelas, puncturing in, improbated ii 27
remark of Mr. Pearson on . xi 327
senile, a malum signum . . xvii 119
description of . . . xvii 118
treatment of . . . xvii 118-9
stimulant applications to . xiii 381
stimulant treatment of . . xi 325
strictures on, Dr. Williams on iii 148
sudden disappearance of . xiii 377
suppurative, Mr. Travers on . ii 24
Sydenham's treatment of . v 366
symptoms of, asthenic . . ii 26
terminations of . . . xi 323
theory and treatment of . . vii 252
tincture of iodine to . . xiii 382
tonic treatment of . . . iii 147, 459
tonics and stimulants in . . xiii 379
treated by bandaging . . iii 558
with cotton . . . v 244
treatment of iii 109, v 368, 608, xi 324
in America . . . iii 459
Mr. Travers on . . ii 26
use of aconite in xi 327, xiii 380, xx 468
ammonia in . . iii 149
emetics in . . . xiii 379
iodine in . viii 521, xiv 563
ipecacuanha in . . xi 327
narcotics in . . . xiii 380
purgatives in . . xiii 379
turpentine in . . xiii 380
venesection in . . . xiii 378
vesications in . . . xiii 376
water as a sudorific in . . xiii 380
Erythema, mercurial, remarks on . v 23
Erythema papulatum, syphilitic . xxiii 354
Erythrosis, or plethora arteriosa . iii 212
Escharotics, observations on . . xvi 196
on the employment of . . xxii 368
Eschricht, Dr., on nutrition of fœtus ix 2
Esdaile, Dr., his cases sifted . xxii 479-85
on mesmerism in India . . xxii 475
Esoteric periods, causes of . . xviii 171
Esoterism, medical, remarks on . xxi 450
Esquimaux, Asiatic traits in the . xxiv 477
mode of cramming children by ii 383
Esquirol, M., his tables of insanity i 273
M. Pariset's eulogy on . . xxi 408
on lunatic asylums . . ix 144
on mental maladies . vii 1, 4, xx 520
portrait and library of . . xix 611
remarks on his writings . . xix 611
Essays, Miss Martineau's . . xviii 472
Essig-ether wine, formula for . xvi 220
Estlin, Mr., on vaccine virus . vi 591
Ethereal oil of turpentine in typhus xxii 473
Ether, alarming effects from inhaling xiii 311
anæsthesia from, discovery of . xxiii 309
effects of, anticipations of . xxiii 548
essential points in inhaling . xxiii 551
evaporation of, on hernia . xv 42
excitement from . . . xxiii 552
hydrocyanic, account of . . ii 208
inhalation of, M. Flourens on . xxiii 572

Ether, inhalation of, M. Longet on . xxiii 570
Dr. Snow on . . . xxiii 573
Mitscherlich on its production ii 190
œnanthic . . . . viii 330
operations made painless by . xxiii 309
production of . . . xi 144
rubbed on hands, smelt in lungs viii 351
Ethers, Mr. Brande's remark on . iv 110
Etherization, apparatus for . xxiii 569, 575
conditions prohibiting . . xxiii 576
danger from . . . xxiii 564
depressing effects from . . xxiii 574
disadvantages of . . . xxiii 565
discovery of . . xxiii 548, 576
effects of, on functions, &c. . xxiii 553
fatal cases, in relation to xxiii 557, 563
in medicine, remarks on . . xxiii 566
in midwifery . . . . xxiii 567
in surgery, remarks on . . xxiii 556
its effects on the blood . . xxiii 555
physical changes from . . xxiii 554
recovery from . . . xxiii 576
the pulse in, remarks on xxiii 553, 574
Ethical judgments of men . . xxiv 227
relations of medicine . . xix 121
Ethics, medical, in the United States iii 276
treatise on . . . xxi 438
Etiology, general . . . xxiv 298
remarks on statistical . . ix 351
Etiquette, medical, treatise on . x 215
Eucharius Roesslin, his midwifery xiv 84
Eunuch, case of a natural . . viii 576
Eupatorium perfoliatum in influenza xx 231
Euphorbia peplus, noxious to hemp iv 130
Eupœdia, or letters to a mother, &c. ii 221
Europe peopled chiefly from Asia . xxiv 465
European branches of Indo-European stock . . . . . xxiv 458
Europeans in India, health of . xii 423
Eusebius on plague in Alexandria xxiv 248
Euskaldunes or Biscayans . . xxiv 464
Euskara, or Euskarian language . xxiv 464
Eustachian canal, tubes for the . iii 94
tube, accidents in catheterizing viii 100
air-douche of, decried . xiv 250
aqueous injections into . iii 95
bad modes of injecting . iii 96
catheterism of . . viii 69, 93
catheters for the . . iii 95
causes of lesions of . . viii 96
Dr. Deleau on . viii 69, 71, 95
guttural orifice of . . viii 76
incurable disease of . iii 97
injection of air into . iii 95
length and width of . viii 75
obstruction of . . iii 96
strictures of . . . viii 97
use of . . . . . viii 87
Eutrophics, a new term for alterative xvii 243
Evans, Dr., his lectures on phthisis xxii 85
Evans, Mr. C., on copaiva . . vi 558
Evans, Mr. S., on obstruction of the large intestine . . . xxiii 411
Evans, Mr. W. J., on West India fevers iv 160
Evanson, Dr., on diseases of children . . iii 439, xi 198, 210
Everitt, Dr., on stramonium in sarcocele . . . . i 578
Eversion of lower eyelid, operation for vii 406
Evidence, medical, remarks on . vi 122
Evolution, of disease, Dr Marx on . xvi 474
spontaneous, case of . . viii 567
in arm, case of . . xiii 568
of a twin . . . xxiii 283
Exaltation of faculties, morbid . xix 467
Examen rigorosum of Prussian physicians i 296
Examination, clinical, M. Louis on . i 404
medical, in Russia . . . iv 270, 278
medico-philosophical, in Prussia i 296
of females by the rectum . ii 514
patients, methodical . . xx 310
practitioners . . . xix 199
per vaginam, standing . . iv 457
Examinations, medical, in America iii 274
Examiners, of Apothecaries' Company, report of . . . . ii 606
of London University . . viii 304
Examining a patient, method of . ii 476
patients, hints on . . . vi 131
Exanthemata, as counter-irritants . vii 69
in Scotland, statistics of . . xviii 511
iodicum . . . xix 518, 523
of vegetables . . . . iv 19
seat of inflammation in . . xv 381
syphilitic . . . . xxiii 353
the air in relation to (App. to vol.) xi 14
Exanthematous diseases, Dr. Bene on i 12
in children . xxi 471
Excavation of lungs, on signs of . ix 311
Excitabilities, many, in living bodies i 391
Excitability, morbid, cherishing . xii 151
of plants, remarks on the . iv 30
Excitants, Dr. Thomson on . . xvi 360
mental, Dr. Thomson on . xvi 361
with diuretics . . . xvi 201
with expectorants . . . xvi 201
Excitement, Brown's theory of . v 80
communication of nervous . ix 404
mental, in regard to health . iv 60
religious, in America . . iv 58-60
Exciting pleasures, morbid effects of xiv 313
Excito-motor power, Dr. Hall on xvii 173, 175
system, anatomy of . xvii 177
theory, on Dr. Hall's . v 500
Excito-motory function, mistake on xii 99
nerves, opinions on . v 538
phenomena, Dr. Hall on . iii 33
system, anatomy of . xvi 167
general account of . xvi 167
Excoriated breasts, remedy for . v 575
Excrements, typhoid, microscopy of xxiii 503
Excrescences, warty, in the heart . ii 326
Excretion, organs and functions of xix 566
volition in relation to . . v 510
Excretions, and food, relations of . xix 564
by animals and vegetables . v 510

Excretions, elements discharged in . xxii 262
of blood in the . . . xiv 307
of plants . . . . iv 27
power of hydropathy over . xxii 452
Excretory canals, reflex action of . xi 455
Exercise, and habit, in organs . xxiv 300
and rest of mind and body . i 340
bodily, on the instinct of . xxiv 199
remarks on . . . i 356
ill-timed, of commercial men . ii 381
importance of, to women . xi 426
its effect on the pulse . iv 242
its hygienic importance . . i 356
its importance to health . . v 145-6
Sir Alexander Crichton on . xv 460
Exeter, medical topography of . vi 382
on the mortality of . . xx 523
Exhalation, cutaneous, in disease . xx 312
cuticular, Dr. A. Combe on . i 330
fluid and true secretion . . xiv 564
Magendie on . . . . viii 317
of carbonic acid . . . xvii 257
of oxygen and carbonic acid . xxiii 395
pulmonary crisis by . . i 246
pulmonary, Tiedemann on . i 241
Exhalations, putrid, effects of . xi 9-13
Exhausting diseases, Sir J. Eyre on xx 203
Exhaustion, of an organ . . xxiv 299
reaction in . . . . viii 527
Exhilaration and depression, states of xxiii 221
Exhumation of bodies at Paris xi 15 (App.) 39
Existence, Cuvier on the conditions of v 88
Exocardial and endocardial sounds . xx 178
Exochorion, villi of the . . . ix 26
Exophthalmia, case of . . . xiv 557
Exophthalmus, from atheroma, case xiv 233
Exosmosis and endosmosis . . viii 317
Exostosis of female pelvis, rarity of xii 490
not venereal, remarks on . xv 51
of pelvis, and embryotomy . vi 237
Professor Albers on . . xvi 382
with osteitis . . . ii 537
Expatriated omentum, Dr. Parrish on iii 362
Expectant system, reaction from . xxii 562
treatment defined . . xv 406, 409
Expectation of life of medical men . i 202
Expectorants, endermic use of . v 351
observations on . . . xvi 194
with excitants . . . xvi 201
Expectoration, black, questions on . iv 150
cryptogamic plants in . . xxiii 510
in phthisis, remarks on . . xxiii 435
of gray firm pellets . . xxiv 496
on black . . . . iv 150
tubular, from bronchi . . xx 96
Expectorations, comparative ease of iv 299
Experience, and practice, remarks on xxiii 157
medical, Dr. Cowan on . . i 170
practical knowledge from . iv 132
unmasked . . . . xx 461
Experimental investigations . . v 332-4
Experimentalists, French, cruelty of xxiv 398
Experimenters, blunders of . . x 508
Experimenters, counsel to . . vi 476
Experiment and reasoning, remarks on viii 352
definition and use of . . v 322
in physiology, remarks on . v 336
value of, in medicine . . xxiii 514
wrong French ideas of . . xxiv 397
Experiments, medical, question on xxi 452
negative and positive . . xiii 480
on air in the veins . . . vi 517
on animals, fallacy of . . viii 149
on painful . . . xviii 344
on living animals . . . viii 310
on some objectors to . . xxiii 162
painful, on animals . . x 185
Experts, in medico-legal cases . xii 175
Expiration, a stethoscopic sign of value i 481
augmented . . . . . ix 310
auscultatory characters of . ix 310
Dr. Harlan on . . . . ii 518
duplex act of, cause of . . xxiv 176
early detection of tubercles by i 481
greater in tall persons . . xxiv 328
two sounds in . . . . ix 304
Expiratory murmur, account of the ix 301
sound, diminution of . . ix 310
Exploration, obstetric, by sight . i 98
by touch . . . i 104
treatise on . . . i 85
of tumours, mischief of . . xv 33
Mr. Liston on . . xxii 374
of a patient, external . . xx 311
Explorator for chest, Dr. Babington's xviii 343
Expression, anatomy and physiology of xviii 442
cases of loss of power of . xi 458
facial, the nerves of . . iv 212
Extase, remarks on the state of . iii 328
Extirpation of the eye, Dr. Bonnet on xv 244
Extractum graminis, use of . . xix 241
Extravasation, in injuries of head . xv 172
Extra-uterine fœtation, anatomy of v 254
gestation, memoir on . x 247
pregnancy, case of ii 570, iii 245, iii 522, xxi 419
Dr. Heim on . . iv 493
gastrotomy in . . xiii 545
in 70th year . . xiv 560
report on . . xxiii 278
Exudation-corpuscles in sputa . xvii 520
Exuded matter, in inflammation of . xviii 271
organization of . . xviii 272
Eye, action of its muscles . iv 477 xii 553
action of the oblique muscles of xi 521
anatomy and physiology of the xix 584
anatomy of the . . . x 9
report on . . . xxii 284
aponeurosis of the . . . xii 258
application of leeches to . xi 58
as an object of medical police iv 480
auricular remedy for . . x 40
belladonna, in diseases of . x 8
blood in anterior chamber of . xviii 421
blood-vessels of, Van der Kolk on xviii 49
books on diseases of, making . xvii 233

Eye, burns of, by gunpowder . . ii 267

cases of severe blows on the . v 37

catoptrical examination of vi 231, 274, x 24

exploration of the . . x 8, 24

cells of membranes of the . ix 510

ciliary processes, diverticula to xxiii 142

curious experiments on the . vi 210

destruction of, after smallpox . xvii 234

by diseases of the heart . ii 333

detached, diagnosis of right and left x 10

diagnosis of tumours of the . x 38

disease of . . . . . vi 74

aqueous membrane of . xi 70

diseases of . . . . . x 20

appendages of . . xviii 413

British schools for . . x 7

castor oil in . . . xv 498-500

counter-irritants in . . xi 58

Dr. J. C. Hall on . . xvi 270

Dr. Little on . . v 31, 45

Dr. Mackenzie on . . ix 543

Dr. Rosas on . . . v 31, 33

galvanism in . . xii 242, 542

in animals . . . xxiv 90

mercury in . . . . x 8

Mr. Jeaffreson on . . xviii 405

nitrate of silver in . . xvii 234

prussic acid in . . xv 497

Dr. Franz on preserving the . x 39

Dr. Werneck, on anatomy of the vi 205

drawings and figures of . . x 20

dry warmth by bags to . xi 58

encysted tumour of . . xviii 420

entozoa in the . . . xviii 422

examination of diseased . x 23

extirpation of, improved mode of xv 244

fungus hæmatodes of, extirpation of . . . . . xviii 420

Galen's disquisition on the . xv 453

general remarks on the . . v 32

greenish hue in, is glaucoma . xvi 112

hair in posterior chamber of . viii 256

hairs within the . . . xii 549

healthy and morbid anatomy of xiii 503

Hofmayer's wax models of . x 21

human, luminous appearance of xxiv 330

inflammation of, Mr. Jones on xx 273

phenomena from . . xx 283

Van der Kolk on . . xviii 48

injury of, from strong acids . xi 65

in relation to diagnosis . . xix 367

internal inflammation of xvi 323, xviii 48

its abdominal sympathies . v 33

its affinities with other organs . v 33

its connexion with the brain . xxi 165

its sympathy with generative organs . . . . . v 34

lacerated, on treatment of . xvii 236

local applications to the . . xxi 163

loss of adaptation of . . xi 86

luscitas of, cure of, by operation xi 482

macula lutea of the . . x 254

membranes of the . . . vii 218

Eye, mobility of, mode of preventing vi 57

mode of applying drops to . xi 57

lotions to . xi 57

ointments to . xi 58

morbid, Dr. Hull on the . xi 221

morbid sensations in the . xx 285-87

Mr. Middlemore, on diseases of the ii 342

on the . . x 3, 22

Mr. Morgan, on diseases of the viii 229

Mr. Tyrrell, on diseases of the xi 55

Mr. Walker's philosophy of . iv 496

muscles of . . . . . vii 457

natural cures of diseases of . ii 270

nerves of, Van der Kolk on . xviii 48

numerous treatises on . . x 1-20

physiology of . . . . . x 13

pupil of, sphincter muscle round xix 584

quinine in diseases of . . x 8

relation of non-luminous rays to xxii 287

report on . . . . . xvii 269

respiratory sympathy of . . v 33

scarifying . . . . x 32

seat of sensations of . . x 15

section of muscles of, in the horse xii 251

singular, of the anableps . vii 228

study of, Sir J. Herschel on . xii 218

of diseases of . . x 3

treatment of diseases of . . viii 231

tumours of . . . . xxii 31

Turnbull on diseases of . . xv 494

turpentine in diseases of . . vi 533

venereal diseases of . . x 393

use of boiling water in dissecting vii 218

warm fomentations and steam to xi 57

works on venereal diseases of . x 381

Eyes, apoplexy of . . . . ix 549

chromatic instruments for . xvi 363

coloured glasses for . . xvi 363

diseases of, caustic to head in . vii 65

fatigue of, operation for . . xiii 238

hysterical affection of . . xiv 246

single vision with two . . x 16

sympathetic action of . . x 33

Eyeball, diagnosis of enlargement of x 585

in relation to vagus nerve . viii 594

muscles and nerves of, Mr. Walker on ii 600

movements of . . . xi 283

Eyelashes, transplantation of . . xxi 310

Eyelid, chalazion of . . . xviii 420

eversion of lower, operation for vii 406

granulated, Mr. Tyrrell on . xi 62

its closure to foreign bodies . v 513

molluscum of . . . xviii 420

under, mode of forming new . xxi 309

Eyelids, anatomy of . . . v 217

adhesion of, to the globe . xx 134

cancer of, cured . . . iii 518

cilia and hair-glands of . . v 217

erysipelas of . . . . x 25

treatment of . . x 25

eversion of, operations for . x 27

formation of new . . . ix 559

with *plate* . iv 483

Eyelids, horny excrescences on . xii 248

inversion of, operations for . x 28

malignant pustule of . . x 26

motions of . . . . . xx 133

obstinate closure of, case of . xiv 246

of young animals born blind . xix 585

plastic operations on . xix 409, xxi 308

restoration of . . . vii 404

sebaceous follicle of . . xviii 420

skin of . . . . . . . v 217

sunken, remedying . . vii 411

wounds of, insect-needles in . xxi 308

Eye-glass, an absurd implement . xi 57

Eye-washes, mode of applying . xi 57

*Eyr*, a medical journal, Danish . i 228

Eyre, Sir J., on exhausting diseases xx 203

Face, and cranium, development of . xviii 446

atrophy of, new form of . . xxiv 43

expression in . . . . . xviii 447

malignant diseases of . . vii 143

muscles of, expression by . xviii 450

of negro, observations on . xviii 446

paralysis of, myotomy in . xiii 241

partial paralysis of . . . v 281

physiognomical rugæ of . . xx 313

-presentations, causes of . . viii 258

in labour . . ii 78, xix 69

in 16,434 labours . . ii 72

Mad. La Chapelle on . xiv 372

management of . . xii 476

natural delivery in . . xii 474

observations on . . xix 424

remarks on . . . viii 47

to the groin . . . xii 472

removal of the bones of . . vii 250

spasms of, myotomy in . . xiii 244

Facial angle of Camper, Sir C. Bell on xviii 446

nerve, Bellingeri on . . ix 143

physiology of . . . xxii 280

nerves, paralysis of . . xiii 235

neuralgia, Sir B. Brodie on . xxii 170

paralysis, agheustia in . . xxii 282

case of, by Dr. Watson . xvii 133

emetics in . . . vii 247

observations on . . xvii 133

spasm, cured by myotomy . xiii 22

Facies Hippocratica, remark on . ii 425

Factories, and mills, report on . xv 285

Austrian, economy of . . xviii 149

climate of, Mr. Mayo on . . v 380

cotton, Dr. Ure on . . xv 293-4, 301

diseases in . . . . . xv 300

domestic accommodation in . v 386

employing children in . . xiv 457

Mr. Noble on the health of . xvi 507

Mr. Wing's testimony on . v 387

scrofula in . . . . . xxii 148

silk, unhealthiness of . . xx 103

state of females in . . . v 387

superior ventilation in . . xv 293

Factory girls, catamenia in . . xv 308

Factory, medical contracts . . xv 332

people, Austrian, healthiness of xviii 151

degeneracy of . . . v 386

population of Manchester . xiv 458

system, Dr. Blundell on . . xv 290

Dr. Elliotson on . . xv 291

Dr. Hodgkin on . . xv 290

Dr. Young's evidence on . xv 289

effects of, on health xv 288, 300, xviii 147

evidence of Dr. Farre on . xv 292

medical evidence on . xv 288,301

Mr. Green on . . . xv 291

Mr. Malyn's evidence on . xv 289

one-sided evidence on . xv 292,313

Sir A. Carlisle on . . xv 291

Sir W. Blizard on . . xv 291

Facts, comparable, in medicine . xii 12, 21

compound . . . . . xii 13

different kinds of . . . xii 12

false, the bane of medicine . vii 117

in medical science, remarks on . xii 13

in medicine, observations on . xxi 509

mode of collecting . . . xiii 470

wanted in medical science . xii 21

Faculties, natural or nutritive . . xv 445

Faculty, advantages of one medical . xiv 410-11

the discriminative . . . xv 446

Fæcal effusion in wounds of intestines xxiii 4

tumours . . . . . ix 447

caution respecting . . x 48

vomiting for thirty-four days . xiv 579

Fæces, chemistry of . . . xvii 435

Dr. Schultz on the nature of . xvi 224

human, analysis of ashes of . xix 277

microscopic constituents of . xxi 564

non-existence of bile in . . xxi 561

perplexing collections of, in women ii 511

typhoid, microscopy of . . xxiii 503

Fætid breath, blundered case of . v 139

Fætid irrigations around Edinburgh xi 1, 19

Fætor, mercurial, cause of . . v 139

Fainting, curious case and cure of . v 389

fever of Persia . . . xvi 558

fits, case of slow pulse in . xiv 55

in relation to the brain . . xv 420

Fairfield (U. S.), College of Physicians, &c. at . . . ii 588

Faith in medicine, remarks on . xxiii 469

in the future, remark on . xxiii 545

Falconer, Dr., his Fossil Zoology . xxi 476

Falck, Dr., on endemic bronchocele xx 168

Fallacies of the Faculty, Dr. Dickson's xv 124

Fallopian pregnancy, cases of xx 527, xxiii 278

tube, bony tumour of . . xi 503

case of hernia of . . x 267

pregnancy of . vii 562

tubes, ciliary motions in . . i 510

Fallot, M., on feigned diseases, &c. xviii 117

Falret and Voison, MM., their asylum xix 608

Falret, Dr., in the Salpêtrière . xix 282

on hypochondria . . . xx 368

Families, difference of children in . iii 473

Family, diseases in children of a . viii 487
likenesses . . . . vii 375
peculiarities, psychical . . vii 377
physicians, duties of . . xxi 506
Famine, and fever, in Ireland . . xxiv 285
Egyptian, cannibalism in an . xix 534
Fanatical physicians, remarks on . xxii 122
Fanaticism, epidemic, nature of . xvii 422
or ultraism, in America . . v 409
religious, in America . . iv 59
Fangs of venomous serpents . . xx 401
Fantasia from a dose of hachish xxiii 221, 223
FARADAY, Mr., on chemical manipulation xv 538
Farcinoma, poison of . . . xiii 97
Farcy, acute and chronic, in the horse xiii 31
acute, by inoculation . . xiii 35
in man . . . . xiii 34
button-, in man . . . vi 119
causes and seat of . . . xxii 48
chronic, in man, doubtful . xiii 35
ending in acute glanders, case of viii 552
-glanders, in man . . . vi 119
in man . . . . . . vi 114
cases analogous to . vi 121
Mr. Percival on . . . xxii 47
FARDEL, M., and the microscope . xvi 18
on softening of the brain . . xvi 1, 33
Farina hordei preparata, formula for xiii 87
Farming work, total abstinence in . xxiv 537
FARR, Mr., his British Medical Almanac iii 206
his Medical Almanac . . vii 294
new bills of mortality . ix 582
reports . . . . . ix 344
statistical reports . . xv 75
on English lunatic asylums . vii 2, 19
on the causes of death . . xi 423
duration of disease . . iii 563
laws of cholera . . vi 576
on vital statistics . . . xviii 197
Far-West doctors, reputation of the iv 194
Fasciæ, direction of abscesses by . x 54
the seat of permanent contractions iv 169
Fasting, in insanity, hurtful to mind ii 548
in the insane, treatment of . ii 548
fatigue and repletion, effects of vii 123
Fat, accumulation of, case of . xvi 386
as a therapeutical agent . xix 369
-cells, structure of . . xiv 263
deep walls of, in operations . xv 35
deposition of on bone . . xxiv 397
remarks on . . . xvii 442
digestion of . . . . xxiv 277
formation of . . . . xxii 261
from exorbitant potation . xvi 197
functions of, in animal frame . xi 355
human, Parisian trade in . v 454
uses of . . . v 454
in food, 'causing bile' . . xvii 32
morbid symptoms from . xvii 32
reconverted into fat . xiv 443
in the system, production of . xiv 508
observations on . . vii 383, xxi 494
of mutton, acids in . . xvii 32
Fat, origin of . . . . . xiv 305
experiments on . . xxiii 393
production of, in the body . xix 564
source of, in animals . . xvii 14
Fattening of animals, remarks on . xiv 306
Fatty degeneration, of arteries . xviii 366
of muscle . . . xiv 543
of the liver . . xiii 553, xxii 28
Fatty, granular, atrophied liver . xxiv 126
Fatty heart, observations on . . vi 48
kidney, interesting case of . xv 488
matter, elaborated by cells . xv 279
matters, in sputa . . . xvii 521
principles, in plants and animals xix 251
substances, nutrition from . xiii 254
tumours, Sir B. Brodie on . xxii 171
Fatuity, cases of recovery from . vii 10
Fauces, morbid anatomy of . . xi 411
peculiar affection of . . v 564
Fauna antiqua Sivalensis . . xxi 476
FAUSSET, Mr., on pulsations of the aorta iv 533
FAUVEL, M., on bronchitis of children xii 350
FAVELL, Dr., on grinder's rot or asthma xxii 320
Favus, confusion on the subject of xv 121
fungus, description of . . xxiii 509
M. Rayer's description of . ii 152
of M. Rayer, rare in England . ii 153
of the scalp, diagnosis of . xv 122
prognosis of . . . xv 123
treatment of . . xv 122
on removing the hair in . xv 122
on the pitch-cap in . . xv 123
seat and nature of . xiii 558, xv 121
FAYE, Dr., on the seminal vesicles xv 346, 361
Feathers, minute structure of . ix 511
Feather white wine, poisoning by . xiv 307
Febricula, sources of (App. to vol.) xi 28
table of convalescence in (App. to vol.) xi 33
pulse in (App. to vol.) xi 33
Febrifuges, experiments on . . xx 238
Febrile blood, reduction of solids in xvii 143
Febris algida, extinction of pulse in xiii 98
cephalica, in children . . xxi 383
hæmatodes, characteristics of . viii 441
nervosa, morbid appearances in i 7
Fecula of plants, nature of the . iv 26
Feculent discharge from umbilicus xviii 340
Fecundation, disquisition on xvi 520, 556
on the act of . . . xv 264
Fecundity, deterioration from . xv 339
of women in France, duration of xx 138
Feelings, of pleasure and pain, seat of xxii 512, 514
sensorial, ideas cerebral . xxii 512
Fees, annual, in Prussian colleges . i 296
for medical lectures in the United States . . . . ii 593
from assurance companies . xiii 578
medical, by assurance offices . iii 54
in America . . iii 575, vii 591
in Bavaria . . . iii 282
in the United States . iii 279
mode of paying . . x 217
question and remarks on taking xxiii 468

Feet, beautiful, of the Madrid ladies iii 153
burning of the, an Indian disease v 131
cure of deformity of . . x 583
delivery by the, Dr. Bush on . v 579
impression of, in limestone . xvii 277
-presentations in 16,434 labours ii 72
superiority of, in Parisian females iii 153
suppressed perspiration of, paraplegia from . . . xxiv 42
well-formed, of the Irish poor . iii 153
Feigned diseases, causes of . xviii 118, 119
classification of . xviii 120, 121
objectionable plan on . xviii 120
prize for essay on . . xviii 120
surgeons' duty in . xviii 122, 123
treatises on . . . xviii 117
Fellows of College of Surgeons, new xvii 283
Female, case of a double . . viii 33, 34
Female accomplishments, remarks on xi 426
animals, artfulness of . . xii 64
diseases, Dr. Ashwell on . xvi 512
from constipation . . x 44
education, absurdities in . xi 426
Dr. Riofrey on . . v 380, 405
form, improvement of the . v 407
labourers in Scotland, state of xi 26
lunatics, employments of . xxi 461
organs of copulation, anatomy of xix 513
puberty, remarks on . . v 408
sensibility and selfishness . v 408
syringe, Dr. Chase's improved v 307
Females, Dr. Churchill on diseases of vi 86, 97
duties of medical men to . xxi 449
examination of, when standing i 105
mortality of, in London . xv 337
rate of mortality less in . . iii 47
responsibility of, in pregnancy vii 129
secretion of milk by aged . vi 80
Feminescence, Dr. Mehliss on . vi 79
Femmes passées, religious meetings of xx 33
Femoral artery, case of rupture of . iii 122
cases of wounded . . iv 155-6
deviations of . . . xix 502
on ligature of . vii 153, xix 504
secured by pressure . iii 124
tying, in aneurism . xiv 132
hernia, complications of . xxii 206
irreducible, pad for . xxii 208
seat of stricture in . xxii 207
muscles, division of . . viii 400
supra-condyloid process . xix 571
Femur, a rare fracture of neck of . xiv 252
bony union of fractured neck of xiv 50
congenital dislocations of viii 559, xii 259
luxations of . . . x 576
diagnosis in fracture of neck of xi 262
dislocation of on pubes, case of viii 286
excision of upper end of . xxiii 415
fracture of neck of . xiv 39, xxiii 419
luxations of, on the pelvis in . iii 240
remote effects of fracture of . iii 238
resection of head of . . xxii 83
separation of epiphysis of . viii 279
Femur, spontaneous luxations of . xiv 554
unreduced dislocations of . xiii 420
Fencing, Dr. Kilgour on . . i 357
Fenger, Dr., on the health of labourers xiv 111
Fenner, Mr., on pressing the uterus vi 567
Fensham, John, apothecary, in Russia, 1593 . . . i 599
Ferguson, Dr., on cachexia Africanorum iii 217
on malaria . . . i 349
puerperal fever . vii 482, 500
the origin of malaria . iv 161
Fergus, Professor, obituary of . vi 289
Fergusson, Mr., his Manual of Surgery xxi 483
his Practical Surgery . . xv 188
surgery, defects in . . xv 188-9
praise of . . . xv 191
on cleft palate . xix 415, xxiii 411
lithotrity . . . vii 286
morbus coxarius . . xxiii 415
Fergusson, Dr., his Notes and Recollections . . . xxii 551
Fermenting bread, loss of gluten by xiii 489
Fermentation, C. Latour's theory of xi 445
in stomach, remark on . . xvi 222
metabolic force in . . ix 523
nature of . . . ix 579, xi 443
of food . . . . xvii 33
Fermented liquors, abolition of, in navy . . . xxiv 532-35
in pregnancy and lactation xxiv 543
inutility of, in field labour xxiv 536-38
in warm climates . . xxiv 530
on drinking . . . ii 388
therapeutic use of . . xxiv 542
use of . . . . ix 243
Fernandez, Mr., his mode of lithotrity iii 266
Ferrall, Mr., on inflamed tumours of cæcum . . . . i 501
Ferrarese, Dr., on madness . vii 1, 50
Ferrario, Dr., on mortality in Milan Hospital . . . xxiv 379
on sudden deaths . . . x 420
Ferrers, Earl, criminal case of xvi 89, 103, xxiv 232
Ferruginous pills, preservation of . xiii 254
Ferrus, M., account of . . xix 602
Fever, acclimating, none in India . xxiii 111
acute, secondary bronchitis in . iv 303
affection of brain in, nature of xvi 234
African, and yellow, identity of xi 369
cause of . . . . xvi 506
Dr. M'William on . . xvi 259
Dr. Pritchett on . . xvi 505
treatment of . . . xvi 505
west coast, nature of . xi 368
agency of marshes in causing . viii 216
a healthy process . . . xv 411
alcoholic fluids in, remarks on xvi 361
alcohol when useful in . . xxiv 544
algide intermittent . . viii 62
American remittent, Dr. Currie on xvii 365
among soldiers and sailors . xv 9
and ague, distinct nature of . ix 467

Fever, and dysentery, at Chusan . xxii 181
and famine, in Ireland . . xxiv 285
and inflammation, distinction of i 30
ataxic, of Prost . . . xii 296
at Bona, treatment of . . viii 58
at Limerick, Dr. Geary on . iv 536
atmospheric, causes of . . viii 445
bilious and typhoid, identity of viii 433
bilious, case of . . . viii 433
remittent, diagnosis of xvii 362, 364
-bill of Dundee, 1833-1839 . xv 341
-blood, characteristics of . viii 441
Dr. Kehrer on . . viii 428
bloodletting in . . . xxiv 2.4
history of . . i 44
brain affection in, treatment of xvi 237
British and French, compared . xii 322
var. of same spec. xii 326
British works on . . . xvii 361
calor mordax in, electrical . viii 445
cases of, treated by cold water xxiii 269
cause of local affections in . viii 332
causes of . . . . . v 270
caution in the treatment of . v 190
cerebral excitement in, respiration in xvi 236
chloride of oxide of sodium in . v 563
clay soil in relation to . . vi 13
cold affusion in, fallacy on use of xxii 447
cold lotions to head in . . xvi 240
comatose intermittent . . viii 62
congestive nervo-bilious . iii 347
of America . . xvii 364, xx 236
of India . . . . . iii 351
considerations on the nature of v 189
contagious, causes of . xi 18, 22, 36
generated anew . . xi 4
in Edinburgh . . xi 27
with dysentery . . xxiv 355
continued, a disease of the blood xii 326
in Ceylon . . . xv 4
in United States . . xvii 357
local affections in . . ix 470
nature of . . . xi 2
not an essential disease . xii 326
not a local disease . . xi 2
of Armstrong . . . xvii 362
of India, described . . iii 349
origin of . . . xi 24
sources of . . xi 2, 20, 24, 36
convalescence in, precepts on xvii 370
costliness of, to towns . . xv 340
critical days in . . . xii 310
cure of, by nature and art . xxiii 587
cutaneous discoloration in . xi 421
delirious intermittent . . viii 62
delirium in, treatment of . xvii 370
diarrhœa in, treatment of . xvii 369
diet proper in . . . xvii 369
difference of typhus and typhoid viii 428
different local affections in . iv 248
distress a cause of . . . xi 24, 28-9
doctrine of, Sir Alex. Crichton on xv 466
dualistic theory, applied to xxiv 136
Fever, Dr. Alison on the cause of . xi 23
Dr. Bene on . . . . i 4
on the treatment of . i 6
Dr. Canstatt on . . . xiii 341
Dr. Clutterbuck on idiopathic . v 187
on treatment of v 189
Dr. Clutterbuck's theory of . v 185
Dr. Gilkrest on yellow . . iv 166
Dr. Graves on . . . xvi 233
Dr. Hodgkin on the cause of . xi 422
Dr. M'Cormac on . . . i 172
Dr. Reed on . . . . xxiii 241
Dr. Stevens's pathology of . xiii 202
Edinburgh, clinical report on . iv 247
effect of acclimatization on (App. to) xi 67
effects of colchicum in . . iv 250
effects of civilization on . . xviii 252
electricity in . . . . viii 445
emetics recommended in . v 189
endemic, in Scotland, treatment of xviii 193
works on xviii 179
entero-mesenteric, of Petit . xii 296
epidemic, in Dublin, in 1826 . xviii 188
in Land's End . . vi 11
in Scotland . . . xviii 492
peculiarities of xviii 182
prevalent in wars . . xi 28
erysipelatous . . . . . xx 237
essential, Dr. Canstatt on . xiii 343
exacerbations in . . . xiii 342
fainting-, of Persia . . . xvi 558
few medicines requisite in . xvii 369
filth, in relation to (App. to vol.) xi 10 57
French doctrine of . . . xv 463
from accumulation of persons (App. to vol.) xi 55
from camphor . . . v 271
from human exhalations (App. to) xi 40
from old army tents . . i 377
from putrid effluvia . . v 271
from putrid effluvia, denied (App. to) xi 37
gastric mercurial inunction in viii 420
sources of (App. to vol.) xi 35
treatment of, by homœopathy xxii 576
general observations on . . xviii 179
geological causes of . . xx 211
Gibraltar, morbid anatomy of . x 496
state of liver in . . x 500
hay-, or summer bronchitis . xx 450
hectic, distilled vinegar in . xix 23
Dr. Clutterbuck on . . v 188
history of, in various climates xv 4
hospitals in Ireland . . . ii 185
hydropathic treatment of . xviii 195, xxii 439, 447
identity of typhus and typhoid (App. to vol.) xi 73-80
idiopathic continued, only one kind of . . . . xvii 362
inanition in, artificial heat in . xvii 356
in Belfast, statistics of . . iii 268
in Britain and Ireland, table of (App. to vol.) xi 53

Fever, in connexion with destitution xviii 512
Peyer's glands xi 420
increase of, in Edinburgh . xi 25
in different English towns . xi 32
in children at the breast . xx 359
independent of inflammation . iii 347
Indian, native remedy for . iii 352
Mr. Twining on . . iii 347
remittent, account of . iii 349
authors on . xiii 358
bark in . . xiii 358
bleeding in . xiii 358
calomel in . iii 350
mercury in . xiii 358
practice in . xiii 358
treatment of . iii 350
quinine in . iii 350
in Edinburgh, 1838-9 . . xii 314
the five-day . . xviii 187
state of blood in . . xviii 189
urea in the blood in . xviii 190
inflammation of parotid in . vi 138
inflammatory case of . . xv 409
infantile remittent, cause of . xii 95
influence of age on (App. to vol.) xi 65, 77
table of . v 271
of chronic diseases on (App. to) xi 62
delicacy on (App. to vol.) xi 61
fear on (App. to vol.) xi 62
idiosyncrasy on (App. to vol.) xi 59
intemperance on (App. to vol.) xi 60
pregnancy on (App. to vol.) xi 70
sex on . . . . v 271
trades on (App. to vol.) xi 69
treatment of . . . v 272
in hospitals, facts on (App. to vol.) xi 3
injurious effects of opium in . vi 555
in Glasgow, from malaria xviii 506, 509
ophthalmitis after . xviii 192
poverty as a cause of xviii 505
in India, morbid anatomy of . iii 351
in Ireland, mortality from . xviii 399
number of cases of . ii 185
in London, decreased mortality from ix 34
Dr. Bateman on . xviii 181
Dr. S. Smith on . xi 11-13
from malaria . . xviii 503
late prevalence of . xi 32
medical reports on . xi 5
1799 and 1800 (App. to) xi 49
in North America . . . xvi 41
in relation to humidity (App. to) xi 45, 54
in relation to rain, table of (App. to) xi 54
in Scotland, black vomit in . xviii 187
bronzed aspect in . xviii 183
on jaundice in xviii 187, 191
rapid pulse in . xviii 183
relapses in . . xviii 184
in St. Lucia, lesions in . . iv 165
state of organs in iv 165
intermittent, at Bona . . viii 59
at Lisbon . . v 558
chloride of soda in . . ii 553

Fever, intermittent, Dr. Leitao on . v 558
etiology of . . . iii 105
hereditary transmission of xx 211
homœopathic treatment of xxii 576
in Ceylon . . . xv 4
Kramer's theory of . . viii 67
M. Brachet on . . iii 105
narcotine in . . . viii 263
nature of . . . xx 347
on pain of back in . . x 557
relapses of . . . viii 65
remedies in . . . viii 64
spinal pain in . . . viii 67
state of spleen in . xii 93, xx 237
theory of . . . viii 60
transmission of . . xii 246
treated by salicine . . i 571
treatises on . . . viii 56
treatment of . . ii 176, xx 238
tying limbs in . . viii 251
(see Ague.)
various forms of . . viii 61-63
intestinal hemorrhage in, treatment of . . . . . xvii 369
sources of (App. to vol.) xi 35
in the navy . . . . xv 7-10
Vale of Severn . . vi 9
in Tuscan marshes, cause of xxi 208, 210
irritative, from warm fluids . viii 277
jail-, alleged sources of (App. to) xi 42
on whitewashing localities of xviii 196
Mackintosh's opinions on . iii 103
malarial origin of . . . xi 13
malaria does not cause . . xi 22, 36
malignant, at Pali . . . ix 234
marsh, observations on . . xiii 350
treatment of, cases of . xxiii 266
medical controversy on . . xv 335
miliary or sweating . . xx 232
epidemic . vii 244
morbid changes in, cases of . xii 315, 321
most fatal local affections in . iv 249
Mr. Stallard on the treatment of xxiii 269
neglect of calomel in, in Paris ii 63
nervous, influence of seasons on xxiv 110
of Huxham, nature of . xvii 362
non-identity of French and English . . . . xii 319, 322
obscured by Cullen's division . ii 55
observations on . . . ix 467
of growth, Prof. Reich on the . ii 219
of Texas, and Gibraltar, compared xii 434
on oil or grease protecting from (App. to vol) xi 72
on the Niger expedition . . xvi 259
on the blood in the head in . iii 324
opinions of the Hindoos on . xxiii 542
origin of, note by Editor on the xi 36
Parisian, inferences respecting xii 304
paroxysmal, in India, treatment of xxiii 117
peculiar epidemic, in Scotland xx 233
petechial, Dr. Peebles on . viii 436
plan of treatment in . . xvii 369

Fever-poison, existence of a . . xi 4

nature of . . . xi 21

sources of . . . xi 5, 24

polypharmic, treatment of . xvii 369

possibility of cutting short . ix 471

preventing cerebral symptoms in xvi 235

primary and secondary . . v 186

primary source of common . iv 187

principles for stimulants in . viii 439

prior to menstruation, remarks on iii 351

Prof. Lanza's views of . . xxiii 56

pseudo-convalescence in . . xvii 370

proximate cause of, Dr. Billing on v 203

puerperal, a blood disease . xxi 276

abscesses of vagina in . xiii 110

on alkalies in . . . ii 489

a peculiar and fatal kind ii 95

a peculiar virus in . . ii 485

bleeding in . . ii 488, vii 496

bloodletting in . . ii 92

bluish-red tumours in . xiii 120

Boer's remedy in . . vii 484

cause of ii 484, vii 484, 490, 494

cause of variety in . . vii 492

change in type of . . xxi 276

commencing . . . xiii 105

complicated form of . vii 486

confusion respecting . vii 483

contagious nature of . ii 488

deposits in . . . vii 499

deposits of pus in . . ii 486

dissection after . . ii 483

Dr. Armstrong on . . i 39

Dr. Ferguson on . . vii 482

Dr. Gooch's opinion on . vii 492

Dr. Gruber on . . vi 539

Dr. Lee's division of . vii 493

Dr. Litzmann on . . xxi 275

Dr. Meigs on . viii 49, xxi 92

dry air and ventilation in ii 488

effusions in . . . vii 489

emetics in . . vii 497, 499

epidemic . . . vii 494

epidemic causes of . . xiii 109

eruptions in . . . xiii 120

existence of, denied . xiii 101

exudations in . . xiii 114

fatality of complicated . vii 487

fetid discharge in . . ii 485

fomentations bad in . ii 93

four forms of . . . vii 483

from morbid blood . . ii 483

gastro-enteric . . vii 485

great mortality in . . vii 495

hot linseed poultice in . vii 485

in Dublin, symptoms of . ii 92

infection of, from practitioners . . . xiii 111

in Paris, 1840 . . xii 536

intestinal irritation in . vii 493

is erysipelatous . . ii 24

its connexion with erysipelas ii 487

leeches in . . . ii 488

Fever, puerperal, leeches to labia in viii 56

lesions of brain in . . vii 489

linseed poultice in . . ii 93

mercurial influence in . ii 92

mercury in . . . ii 489

morbid anatomy of . vii 488

changes in . . xiii 121

Mr. Moore on . . ii 481

M. Tonellé's theory of . vii 493

nature of . . ii 482, vii 490

nervous form of . . vii 486

not from phlebitis . . ii 485

origin of . . . xiii 110

pathology of . . . iii 525

peculiar effects of . . ii 486

peritonitic form of . . vii 485

pneumonia in . . xiii 117

precaution against . . i 11

purgatives in . ii 489, vii 497

purulent deposits in . vii 489

remarkable cause of . xxii 197

remarks on . . . viii 52

report on . . xx 536, xxiii 290

Ritgen's theory of . . vii 493

singular origin of . . ix 571

sporadic origin of . . xiii 102

state of the blood in . ii 483-4

symptoms of . . . vii 484

treatment of ii 92, vii 495, xiii 112

turpentine in . . vii 499

use of chlorine in . . ii 488

value of symptoms in . vii 490

variations of . . . xiii 109

varieties of . . ii 91, vii 483

with abdominal pain . vii 484

(see Puerperal Fever.)

rare in sandstone district . vi 13

rare in slave ships (App. to vol.) xi 41

remarks on symptomatic . . v 186, 188

remittent, at Hongkong . . xxii 182

in Ceylon . . . xv 4

in children, report on . xvii 563

morbid anatomy of . xvii 363, 367

report on cases of . . . xviii 337

reservation of medicines in . xvii 369

rhythm of, Dr. Canstatt on . xiii 342

scarcity in relation to (App. to vol.) xi 46

scarlet, Dr. Graves on . . xvi 241

Schönlein's notion of . . xxi 22

secret of successful treatment of xxii 551

simple, becoming contagious . i 373

spotted, saline treatment of . iv 535

typhus, of Ireland . . xviii 38

state of the blood in . . xx 347

statistical inquiry on . . vi 556

Stevens's saline treatment of . iv 535

sugar of lead in cases of . v 239

support of the system in . xvii 355

table of complications of (App. to vol.) xi 25

table of local affections in . iv 248

tartar emetic and opium in . xvi 238

Texas yellow, morbid anatomy of xii 435

on contagion in . xii 437

Fever, Texas yellow, treatment of . xii 437
theory of . . . . . xx 346
treatment of, by Dr. M'Cormac i 172
delirium in . . ix 473
type of, in the British legion . vi 450
types of, at Bona . . . . viii 57
typhoid, account of eruption in xii 315
adynamic . . . ii 54
age most liable to ii 52, viii 431
appearances after death from ii 42
ataxic . . . . . ii 54
bilious . . . . . ii 53
contagious nature of . viii 430
diagnosis of . . . ii 55
diseases of follicles of intestines in . . ii 42
duration of . (App. to vol.) xi 29
fatality of, in Paris . . ii 56
fatal symptoms in . . ii 39
favorable symptoms in . ii 58
first period of . . . ii 35
in children, report on . xvii 563
inflammatory . . . ii 53
insensible degrees of . viii 434
in Sweden, Dr. Huss on . xxii 469
invasion of . . . ii 34
Louis on . . v 549, xii 22
M. Chomel on . . ii 33
on chlorides in . ii 64-68
contagion in . ii 52
the nature of . ii 68-70
M. Chomel's division of . ii 53
three periods of . ii 35
mucous . . . . . ii 53
not contagious ? (App. to vol.) xi 15
observations on . . xx 235
precursory stage of . . ii 34
primary lesion in the blood ? ii 70
prognosis of . . . ii 56
queries respecting . . viii 434
rose-coloured spots in . ii 36
second period of . . ii 36
signs of perforated bowels in ii 39
slow nervous . . . ii 54
sulphate of quinine in . xv 235
symptoms and progress of ii 34-9
table of convalescence in ii 38
its exciting causes ii 51
third period of . . ii 38
treatment of . . . ix 218
in America . . xvii 368
in France . . xvii 368
its complications . ii 61
its varieties . . ii 59-61
symptoms of . . ii 61
ulceration of intestines in ii 43
unfavorable symptoms . ii 58
varieties of . . . ii 53
typhus, at Frieburg, treatment of xxiv 137
Dr. Bartlett on . . xvii 357
Dr. Lombard on . . iii 260
Dr. Parry on . . . iii 548
Dr. Stokes on wine in . viii 281
Fever, typhus, in relation to glanders xxiii 507
in Scotch cities . . xiv 456
mean duration of . . xii 320
reaction in . . . viii 445
remarks on . . . xi 3
sources of . (App. to vol.) xi 1
state of the heart in . viii 281
table of lesions in (App. to vol.) xi 79
the pupil in . . . vi 554
the rash in . . viii 432, 436
treatises on . . . xii 293
treatment of . . viii 438, 447
by homœopathy . xxii 573
use of digitalis in . . . v 189
emetics in . . . ix 472
hydrargyrus c. creta in . viii 439
ipecacuanha in . . viii 447
opium in . . . viii 439
quinine in . . . xiii 560
utility of alcohol in . . xvii 355
variations in . . . . xviii 182
ventilation in relation to (App. to vol.) xi 11, 57
West India, bloodletting in iv 166-7
calomel, &c., in . iv 167
Mr. Evans on . . iv 160
treatment of . . iv 166, 167
what favour communication of (App. to vol.) xi 57
wine in, new indication for xvi 240
yellow, anatomical lesions in . xvii 367
and remittent, diagnosis of xvii 365
different . . . xvii 368
a kind of, in Dublin . xvi 240
attacks but once . . x 505
colour of the liver in . xvii 367
contagiousness of . . xxiii 427
Dr. Armstrong on . . xviii 223
Dr. Bone on . . . xxiii 426
in Gibraltar . . ix 71, 73, x 494
in British troops . . ix 71
in India . . . . iii 347
in Texas, account of . xii 433, 437
new etiology of . . viii 316
observations on . . iii 106
treatment of . . . xxiii 428
Fevers, acute, continued . . xxiii 61
and exanthemata, treatment of xxii 561
arsenic and quinine in . . xiv 47, 49
circumstances aiding diffusion of (App. to vol.) xi 45
continued, from malaria . . vi 12
not typhoid, sources of (App. to vol.) xi 28
observations on . . xi 1
sources of (App. to vol.) xi 1
difficulty of subdividing . . viii 432
doubtful origin of malignant . xi 370
Dr. Bene's distribution of . i 5
epidemic, of Ireland, history of xviii 39
eruptive, Dr. Mackintosh on . iii 107
on bleeding in . . iii 108
seat of . . . iii 107

Fevers, essential, identity of . . xii 298
from cerebro-spinal lesion . viii 61
in children, reports on . . xvii 561
India, Dr. Geddes on . . xxiii 112
soldiers and sailors . . xvii 317-9
the United States' army . xvi 39
long, less fatal than rapid . i 408
M. Andral on the blood in . xvii 143
Mackintosh's classification of . iii 103
marsh, M. Boudin on . . xiv 43
treatment of . . . xiv 47
minute doses of arsenic in . xiv 47-8
of hot climates . . viii 65, xiv 43
of marshy countries . . viii 65
paroxysmal, diagnosis of . xxiii 114
in India, phenomena of . xxiii 114
periodic, endemic . . . xxiii 61
periodicity in . . . xviii 168
pernicious, intermittent . . viii 61
relative prevalence of . . vi 9
report on . . . . . xxiii 305
sources of, from river malaria (App. to vol.) xi 43
typhoid, alum in . . . i 568
typhus, treatises on . . viii 428
variable types of . . . v 190
Fibre, Dr. Barry on . . . xiii 554
origin of . . . . . xv 234
Fibres, development of . . . xxiii 139
floating, in the blood, Mayer on xiv 295
Fibrilla, muscular, ultimate composition of . . . . . xxi 481
Fibrils, two sets of, in each nerve . xxiv 193
Fibrin, abstraction of, from blood . viii 330
and albumen, difference of viii 348-9, 350, xvi 284
Dumas on . . . xvii 428
identity of . . vi 440, xii 563
L'Heritier on . . . xvii 428
relations of . . . xiv 511
an excrementitious product . xx 72
coagulation of, remarks on . xv 276
change of albumen into . . xv 269
composition of . . xv 229, xvi 284
discovery of a vegetable . . xvi 285
Dr. Polli on . . . . xxi 542
excess of, in inflammation . xviii 257
exudation of, in inflammation . xviii 72
formation of . . . . xx 347
in coagulable urine . . xx 75
inflammatory blood, source of xviii 260
the blood, on reducing . xvii 482
proportion of . . xvii 136
metamorphosis of . . . xx 74
of blood, experiment with . vi 441
organization of, in wounds . xviii 73
quantity of, in the blood . xviii 258
relations of to nutrition . . xvii 482
salts which dissolve, action of xx 75
softening of coagulated . . ix 436
solution of, in the blood . vi 442
structure of . . xiv 572, xv 234
venous and arterial, different . xvii 483
Fibrin, very soluble in acetic acid . vi 440
Fibrinous coagula, substances in . ix 326
concretions in the heart ii 325, viii 273, ix 386
dropsy, Prof. Vogel on . . xxii 325
Fibro-calcareous tumours of uterus ii 165
Fibro-cellular tissue, report on . xvii 251
structure of . xiv 272
Fibrous tissue, contractile, atrophy caused by . . ii 462
development of . xv 277
from pus-cells . . xx 122
tumours of uterus, diagnosis of xxiv 509
remarks on . iv 371-2
symptoms of . xxiv 507
treatment of . xxiv 509
uterine tumour, diagnosis of . vii 451
Fibula, dislocation of head of . xiv 40
Fichte, his philosophical opinions . v 83
Fictitious disease, case of, by Dr. Hall i 204
Fields of Britain, exhaustion of . xvii 226
Fièvre charbonneuse in animals . xxiv 89
Fife, Dr., on creosote . . . vi 270
on pulmonary expectoration . i 242
on watery vapour from the lungs i 242
Fifth nerve, Dr. Alcock on the . iii 257
Figs, proportion of sugar in . . xvii 20
Filiaria medinensis dracunculus . xii 429
Fillet or lacque, in midwifery . xii 486
Fillirea latifolia, use of, in ague . xxi 212
Fillirina, and its sulphate, in ague . xxi 212
Filters, Mr. Thom's self-cleansing . xviii 497
Filth, and fetor, fever from . . xi 411
in relation to fever (App. to vol.) xi 10, 57
Filtration, an effect of, on solutions xxiii 384
Final causes, Mr. Whewell on . v 338
Finck, Dr., on the cure of hernia . vi 341
Finger, contracted, anatomy of a . iv 172
ring fast on, mode of removing vii 515
-talking, by the deaf, remarks on xxi 196
Fingers, and toes, operations on . xxi 330
case of hypertrophy of . . xxiii 413
contraction of, new variety of iv 171
permanent flexure of, cure of . viii 401
retraction of . . . i 269
reunion of divided . . . xiv 231
spasmodic affection of . . iv 507
Finlaison, Mr., his error on female lives . . . . iii 47
Finns, and Lappes, different races . xviii 379
form of their skulls . . xviii 375
skulls of, remarks on . . xxiv 75
Fire-arms, time when fired, determining xvi 73
Fire, French report on cures by . vii 64
Hippocrates on cures by . . vii 63
remedial powers of . . vii 63
the tonic, *par excellence* . vii 63
Fires on the cornea, removal of . ii 269
Fir wood, chemical constituents of. xi 437
Fischer, Dr., his treatment of diseases of the eye . . . iv 481
Fisher, Dr., on cerebral auscultation vii 265
on the cerebellum . . . viii 576
Fish, substance causing brilliancy in ii 451

Fishes, and reptiles, spinal cord of . v 500
attachment of the teeth of . xx 393
brain of, homology of . . xxiv 573
observations on . . xxii 495
development of the teeth of . xx 396
ear of, discovered by Hunter . xv 213
effect of change of medium on vii 176
osseous, on the craniology of . xxiii 483
structure of . . . xxiii 491
pulmonic heart of, remark on . xiv 198
shedding of the teeth of . . xx 397
study of the skeletons of . xxiii 479
substance of the teeth of . xx 394
teeth of, great variety in . xx 391
temperature of . . . xii 144
Dr. Davy on . . . ii 217
Fissiparous generation . . . vii 184
Fissure of the anus, history of . v 484
the incision for . v 485
Fistula, case of intestino-vesical . xiv 134
case of vesico-vaginal . . x 282
hepato-pleuro-bronchial . xiii 236
in ano, incision for . . v 482
in phthisis, remarks on . xvi 434
treatment of . . . v 481
indurations surrounding . xv 42
injection for . . . . xxiii 375
intestinal, from hernia . . xxii 202
lachrymalis, operation for . vii 410
treatment of . . . x 29
large cæcal, in the loins . xi 248
new instrument for closing . vii 142
of ileum and bladder, case of . xx 101
recto-vaginal, cases of . . ii 201-2
renal, M. Rayer on . . xv 477
thoracic, remarkable case of . xiv 556
urethral, suture for . . vii 398
urinary, operations for . . vii 413
vesico-vaginal . . . vii 415
Indian-rubber bottle in . xvii 544
operation for . . . iii 511
report on xvii 543, xx 547, xxiii 297
treatment of . . . iii 427
Fistulæ, and ulcers, Dr. Cramer on i 268
callous, boiling water in . xi 252
fecal, in artificial anus . . xii 460
intestinal, cured by actual cautery ii 567
lachrymal, treatment of . xxi 310
of the larynx, external . . xiv 232
vesico-vaginal, new mode of treating ii 561
treatment of . . xxi 315-20
Fistulous openings, use of iodine in viii 523
Fits, nine-day, in children, Dr. Clarke on ii 97
Fits, prevented by pressing carotids xvii 497
throbbing of carotids before . xvii 497
Fitozoid productions, Dr. Lanza on xxiii 58
Fitzherbert, Mr., his translation of Raciborski . . . i 489
Five children, woman delivered of . viii 564
Flagellants of Italy, account of the . iv 57
Flagellation, religious, history of . iv 57
Flame, inhalation of . . . v 439, 440
Flannel interdicted by Priessnitz . xxii 439
Flannel, in the army, Dr. Ballingall on i 334
its hygienic value . . . i 334
its use in H.M.S. Valorous . i 334
the navy and army . i 332-4
next the skin, Capt. Murray, R.N. on i 334
remarks on leaving off . . i 333
Flap, in plastic surgery, Dieffenbach on xxi 293
-amputation, Sir C. Bell against vi 169
-operation for vesico-vaginal fistulæ ii 563
Flat-foot and valgus, distinction of xiii 9
case of, operated on . xiii 9, 11
causes of . . . xiii 10
characters of . . . xiii 8
from paralysis, operation for xiii 12
operation for . . . xiii 11
prevalence of in Pomerania xiii 10
Flat-head Indians, skulls of the . x 482
Flattening the head, Indian mode of x 483, xviii 224
Flax-pond-water, innoxious . v 451
Fleischmann, Dr., his homœopathic treatment . . xxii 567, 572
on his report . . . xxi 503
homœopathy . . . xxi 240
remarks on his table . . xxii 558
Fleischmann's hospital, cases in xxii 574-92
Fleming, Dr., his treatise on aconite xx 464
Flesh, inorganic constituents of . xxiv 268
juices of, constituents of . xxiv 264
electric current in . . xxiv 269
Liebig on boiling . . . xxiv 271
on boiling of, for soup . . xxiv 271
Fleshy growths on the cervix uteri ii 515
Fletcher, Dr., biographical notice of ii 302
his defects as a philosopher . xv 372
inaccuracy as to facts . v 96
pathology . . . xv 367, 372
strictures on . . xv 368-72
physiology . . . v 75, 92
sarcasms of . . . . xv 370-1
his death . . . . . ii 302
Fleury, M., on consolidation of fractures v 248
Flexion and extension, Dr. Stilling on xvii 399
Flint, discovery on the nature of . iii 582
Flint, Dr., on dyspepsia, from the mind xii 274
Flies, quassia poisonous to . . iv 130
Floating of bodies, remarks on . v 176
of infant's lungs, remarks on . ii 412
Flogging in the army, objections to xxiii 166
responsibility in . . xxiii 165
pleurisy occasioned by . . ix 317
Flood, Dr., his anatomy of the arteries viii 538
Flood, Mr. S., on consumption . xv 540
Flooding, compression of the aorta in x 274
cupping-glasses to mammæ in xxii 156
Dr. Meigs on . . . xxi 91
during labour . . . . x 84
Flora medica, Dr. Lindley's . . vii 531
of Cashmere, by Mr. J. F. Royle i 215
Florence, medical statistics at . xxiv 362
Florida, as a residence for the consumptive . . . . xiii 427
in phthisis . . . xvi 42
pulmonary diseases in . . xiii 427

Flour, British, inferiority of . . xiii 489
improvement of, by peas . xiii 491
FLOURENS, M., his experiments on the nerves . . . . . i 244
his strange experiments on bone . . . . xxiv 400, 402
"his theory" on bone . xxiv 397, 400
on formation of bone . . xxiv 386
inhalation of ether . . xxiii 572
the nervous system . . xvi 364
skin . . . . . vii 239
spinal marrow . . v 532
piece of claptrap of . . xxiv 407
FLOYER, Sir J., his history of cold bathing . . . . . xxii 432
Fluctuation as a diagnostic . . vi 267
Fluid, diagnosis of consistence of . vi 267
Fluids in serous cavities, age of . xii 135
morbid anatomy of the . . vi 298
of the body, remarks on . xxiii 587
vitiated, in puerperal fever . vii 490
Vogel on the mixing of . . xxiv 276
Fluor albus, chronic, cured by iodine iii 509
Fluoric acid in animal substances . ix 396
Fluorine, as an element of food . xvii 4
in bones ascertained . . xix 257
Foci, epidemic, in relation to plague xxiv 254
FODÉRÉ, M., his remark on governments v 448
Fœtal disease, case of . . . xiii 568
heart, auscultation of v 168, 596, viii 368
pulsation of . . . i 89
pulsations of in utero . viii 581
lungs, average weight of . v 180
pulse, remarks on the . . viii 367
when first detected . viii 368
structures, on nutrition in . xx 125
tumour of scrotum, case of . x 255
Fœtation, case of extra-uterine . xviii 340
extra-uterine, cases of . . xxiii 425
Fœtus, amputation of limbs of . . v 614
anchylosis in extremities of a . v 579
and uterus, communication of ix 28
a putrid, graphic description of ii 407
bronchocele in a . . . xi 229
case of croup in . . . xvii 549
cataract in the . . . xi 216
causes of diseases in the . ii 365-6
cephalæmatoma in the . . xi 216
circulation in an acardiac . vii 182
dead in utero, changes in . viii 53
Fœtuses, the circulation of acardiac xiv 198
diseases of the . . . xx 548
classification of . . xi 212
report on . xvii 549, xxiii 298
the lungs in . . . xi 215
their treatment . . ii 365-6
Dr. Graetzer on diseases of the xi 212, 216
enlargement of kidneys in . xi 227
formation of the, Galen on . xv 454
fracture of the bones of . xi 215
full-grown head of, Dr. Naegele on xix 61
hernia and malformation of . viii 594
human attributes and rights of vii 133-5
Fœtuses, injuries of the skull of . xi 216
in utero, Hufeland on diseases of ii 365-6
sounds from limbs of . viii 369
invested by false membrane . xii 348
jaundice in the . . . xi 214
left arm nearly severed in a . xxiii 298
length of, at different periods ii 406
male influence on, in utero? . xiv 240
means of preserving the . ii 366-7
nourishment of, remarks on . viii 45
only an intestinal worm . vii 133
peritonitis in the . . . vii 284
poisoning of, by ergot of rye . xx 528
pulse of, unaffected by mother's viii 367
respiration and nutrition of . ix 2
retardation of growth of the . iii 415
rights of the, on Jörg's opinion of xiv 529
signs of maturity of . . xvi 310
singular skin-disease of . xvii 549
smallpox in the . . . xi 213
spontaneous amputation in . xi 213
of limbs of . iii 267
evolution of . . . iv 405
stone in the bladder of . . xi 214
unaffected by food of mother . iv 449
viable at six months . . xiii 255
without brain or spinal marrow iii 8
heart or lungs, case of . ii 596
Follicles, diseased, in cholera . ii 45
in phthisis . . . ii 46
relation to typhoid symptoms ii 47
scarlatina . . . ii 46
erosive ulcer of the . . iii 556
Graafian, and contents of . xvi 513
formation of . . . xvi 518
intestinal, ulceration of . . xv 95, 97
of intestines, diseases of, in typhoid fever . . . ii 42
Follicular cutaneous disease . . iv 180
disease, causes of . . . xxiv 493
general treatment of . xxiv 504
in typhus . . . iii 260
its value as a characteristic in typhoid fever . ii 70
of larynx, diagnosis of . xxiv 496
pharynx . . . xxiv 485
throat, cases of . . xxiv 486-9
pathological anatomy of . xxiv 492
secondary in fever . . ii 70
symptoms of . . . xxiv 495
tobacco a cause of . xxiv 494, 506
treatment of . . . xxiv 497
with phthisis . . xxiv 491
enteritis, disquisition on . ii 69
in children . . . xvii 560
inflammation, typhous . . xv 95
ulceration, typhous . . xv 95, 97
Folliculi minimi in intestines . . i 526
Fomentations, in puerperal fever, bad ii 93
remarks on . . . . vii 63
Fomites, affected by colours . . i 378
by temperature and distance i 378
conveyed far by clothes . i 377

Fomites, Dr. Henry on . . . i 377
summary of our knowledge of. i 377
Fons vitalis, the brain the . . i 558
FONTANA, his discoveries . . xiv 486
on inhaling hydrogen gas . xxiii 548
Fontanelle, anterior, closure of the xvii 372
uses of the . . . xvii 373
varying size of . . xvii 373
the great, observations on . xvii 372
Food, abstinence from solid, cases of xvii 349
a cheap kind for the poor . xiii 496
action of vital force on . . xvii 5
adulterations of . . . xvii 24
amount of, in relation to age . xvii 42
adaptation of, to assimilation . xvii 38
and diet, Dr. Pereira on . xvii 1, 54
excretions, relations of . xix 564
physic to newborn infants ii 383
animal and vegetable . . xiii 486
on its quick digestion . ii 542
a nitrogenous substance required in . . . . . . xxiii 497
artificial, to children, thrush from xxiv 430
case of ten days' abstinence from xix 565
change from vegetable to animal ii 542
chemical constituents of . xvii 31
chemistry of, Liebig on . xxiv 264
cohesive force of . . . xvii 31
comparative nutritiveness of . xiii 486
conversion of principles in . xvi 481
cookery of . . . . . xvii 34
decayed and putrid, the use of xxiii 160
deficient, convalescence after viii 527
ill consequences of . . xvii 43
digested, on absorption of . xxiii 498
digestibility of . . . xvii 31
Dr. Thomson on . . . xxiv 332
due proportion of liquid in . xvii 41
elements of . . . . xvii 2
disposal of, in the body . xvii 5
experiments on . . . xiii 490
fermentation of . . . xvii 33
importance of analysis of . xvii 29
influence of, on form of stomach ii 541
in relation to climate and season xvii 44
to size of organs . . xvii 40
kinds of, time of digestion of . xiii 493
liquid, absorbed by the stomach ii 377
loathing of, from insufficient diet xvii 348
Mr. Grisenthwaite on . . vii 538
morbid effects from deficient . viii 524
necessity of a variety of . . xvi 224
non-azotized substances in . xiv 516
nutrition from different kinds of xvii 29
from nitrogenized . . xvii 38
nutritive and respiratory . xiv 306
qualities of . . . xvii 38
of animals . . . . . xxiii 492
and plants, Liebig on . xiv 504
observations on xiv 302-5, xxiii 158
treatises on . . . xxiii 157
of cattle, observations on . xxiii 499
children, remarks on . . viii 524
Food, of Cornaro, remarks on . . xvii 41
different nations . . xvii 16
remarks on . . v 410
man, on the original . . xxi 149
nurses, remarks on the . ii 383
the Eastern Christians . xvii 41
labouring poor, Dr. Combe on ii 384
once a day, by a monk of La Trappe ii 381
by the Indian . . ii 381
original, of man, observations on xxiii 496
passage of, into the blood . xxiv 276
prejudices against articles of . xvii 16
preservation of articles of . xvii 34
proneness to acidity of . xvii 33
proper quantity of . . ii 382
proximate principles unfit for . xiii 254
requisite quantity of . . xvii 39
Scriptural arguments on . xxiii 495
supply of, effect of civilization on xviii 242
symptoms from deficient . viii 525
tendency to putrescency of . xvii 34
the sense of a sufficiency of . vi 179
to appetite, advocates for . ii 380
treatment after long deficient . viii 526
various modes of cooking . xvii 36
vegetable, on slow digestion of ii 542
Whytt on its stimulant effects ii 464
Foods, animal, constituents of . xvii 18
observations on . . xvii 18
vegetable, arrangement of . xvii 19
Foot, amputation of the . . vi 535
deformed by short shoes, case of iii 151
dislocation of . . . vi 538
human, treatise on . . . xx 521
pain of, from stricture of urethra iv 133
remarks on the form of . . iii 153
splay-, cause and treatment of. viii 399
susceptibility to impressions of v 519
Foot-baths, cold, for sleeplessness . xxii 54
-Guards, mortality in . . viii 224
-presentation, diagnosis of . i 115
Footling-cases, danger to child in . xix 71
Dr. Hamilton on . . iv 405
FOOTE, Mr., his ophthalmic memoranda vi 202
FOOTE, Mr. Jesse, his Life of Hunter iv 75
Foramen lacerum, contraction of . xx 230
magnum, in the Negro . . xxiv 80
of Bichat, existence of . . xiv 464
ovale, closure of . . . xvii 274
open, old mistakes on . vi 48
without disease . vi 48
FORBES, Dr. J., Dr. M. Hall's wrath at xxiii 203
his illustrations of mesmerism . xxi 277
Medical Bibliography . i 513
trial of Mlle. Julie . . xxi 537
Forbidden remedial means, remarks on xxi 452
Forceps, galvanic obstetric . . viii 566
German mode of introducing . iii 419
in labour, use of . . . viii 49
long, application of . . xii 483
cautions on use of . . xii 485
construction of . . xii 483
injudicious use of . . xxiii 283

Forceps, midwifery, history of . iii 418

introduction of . . xiv 371

Leake's three-bladed . iii 419

mode of applying . . xix 418

Parisian practice with . ii 77

the long . . . xii 482

various kinds of . . iii 418

obstetric, Dr. Ryan on use of . ii 525

straight and crooked . ii 525

on padding . . . . iii 410

remarks on use of . . . iii 410

short, application of . . xii 482

forms of . . . xii 481

use of, in delivery . . . ii 76

Force, disquisition on the term . xi 386

Forces, mutual, convertibility of . xvii 448-9

transformation of, illustrated . xvii 449

vital, Dr. Arnold on . . v 91

Forcke, Dr., on veratrine . viii 263, 353

Fore-arm, in children, dislocation of v 284

treatment of fracture of . vi 309

Foreign bodies, in bowels, cases of . iii 236

in bronchi . . . xx 245

graduates . . . . . xix 226

medical institutions . . i 289

medical journals, account of . i 217

medicine, reports of . . i 289

Forensic medicine . . . iii 271

cases in . . . iii 248, 271

definitions of . . ii 391-2

Devergie and Taylor on . ii 390

Dr. Guy on . . . xvii 228

not taught in France . ii 394

on education in . . x 195

Forseeings, sensational, Unzer on . xxiv 191

Forgetfulness, or loss of memory . vii 9

Forget, M., misinterpretations of . xii 296

on epidemic meningitis . . xv 236

on typhoid fever . . . xii 294

Fork, extracted from the back, case of v 609

Fork-grinders, great unhealthiness of xviii 308

mortality-table of . . xviii 309

Formations, hetero-plastic . . xiii 339

Formative act, Dr. W. Clark on the i 390

and healing effort, the same . xxi 528

globules, changes in . . xxiv 129

process, Prof. Lanza on . . xxiii 51

Formic acid, made by chemical means xi 135

Forms and qualities, propagation of . vii 375

Formula, empirical, of blood . . xiv 514

Formulæ, chemical, remarks on . xvi 478

empirical, of bile and urine . xiv 514

objects in composition of . xvi 198

officinal, complexity of . . xxi 263

promoting the action of basis of xvi 198

Formulary of hospitals, &c., Edwards's ii 210

of new remedies, Magendie's . ii 207

Forry, Dr., on climate . . . xiii 423

strictures on his criticisms . xiii 434

on United States' climate . xvi 34

Fossil anatomy, Mr. Travers on . vi 192

bones, John Hunter on . . vi 192

flora of coal formations . . iv 23

Fossil zoology of the north of India xxi 476

Fothergillian medal for 1837-8 . ii 302

Foundling hospital of Moscow i 606, iv 276

of St. Petersburg xii 273, xxi 466

hospitals, charges against xiii 293, 311

considerations on . . xiii 311

history of . . . xiii 290

mortality in . . . xiii 306

observations on . . xxii 339

open admission to . . xiii 304

the tours in . . xiii 292, 300

treatises on . . . xiii 289

Foundlings, causes of mortality in . xiii 307

effect of suckling or feeding . xiii 308

history of, in Austria . xxii 338, 342

in Austria, statistics of . . xxii 342

mortality of, in Austria . . xxii 344

in France . . xiii 307

numbers of, in France . . xiii 298

perils of, in hospitals . . xiii 310

statistics of, in France . . xiii 289

Foundling system, Roman Catholic and Protestant . . . xxii 345

Foundries, in Manchester, teetotalism in xxiv 335

Fourcault, M., on chronic diseases xx 102

Fourchette, lacerations of . . viii 38

Four masters, their method with divided intestine . . . xxiii 21

Fournet, M., general survey of his work ix 342

on auscultation . . ix 299, xiii 220

therapeutical gambols of . ix 322

Foville, M., at Charenton . . xix 596

on anatomy of the brain . . xviii 423

base of the brain . . xv 231

structure of the brain . x 296

Fowl and duck, temperature of . xii 143

Fowler, Dr., on the deaf and dumb xvii 242

Fowler, Mr., case of molluscum by ix 221

Fowls, pepper poisonous to . . iv 130

Fownes, Mr., his Manual of Chemistry xix 245

his prize essay . . . xviii 531

Foxes, badgers, &c., use of, as food . xvii 16

Foxglove, account of, by Dr. Johnstone i 193

case of poisoning by . . xviii 564

Fox, Mr., case of intussusceptio by . ix 221

nonsensical notions of . . viii 544

on chlorosis . . . . . viii 542

Fractured bones, on rebreaking . xx 118

limbs, on position of . . xiv 9

watchfulness in . . xiv 9

ribs, displacements of . vii 556, 559

keeping immoveable . vii 558

reducing . . . vii 558

treatment of . . . vii 558

Fracture, and dislocation, of ankle . xiv 41

Fracture-apparatus, moistening . xiv 10

oil varnish to . . . xii 544

-bandage, immoveable . . vii 254

-bed, new, of Mr. Greenhow . vi 582

Fracture, callus long pliable in . xiv 10

complicated with wound . . xiv 9

compound, pus in . . . xiv 10

dislocation with, reduction of . xiv 32

Fracture, formation of callus in . x 357

longitudinal . . . . . xiv 7

meddlesome surgery in . . xiv 10

of base of the cranium . . xv 173

carpal end of radius . . ii 256

child's arm, in labour . . x 577

cranium, by contre-coup . xv 173

femur, remote effects of . iii 238

fore-arm, treatment of . vi 309

humerus, ununited, cured . vi 69

inner table of the skull . xv 174

leg, cause of death from . iii 271

lower end of radius xiv 230, xv 242

neck of femur, rare ii 159, xiv 252

of femur, diagnosis in . xi 262

of thigh-bone . . xiv 39

radius, remark on . . vi 309

ribs . . . . . . vii 554

by contre-coup . . v 574

causes of . . . vii 554

incomplete . . . vii 555

Lisfranc on . . . xiv 11

multiple . . . vii 556

simple, complete . . vii 556

spontaneous, case of . vi 227

skull, compound, case of . v 177

during delivery . . iv 96, 233

flux from ear in . . xv 174

punctured . . . xiii 133

starlike . . . xiii 133

through the orbit . xv 178

spine, cured by extension . xvii 98

thigh-bone, gradual extension in iii 130

Josse's apparatus for iii 128

on applying leeches to . . xiv 4, 9

partial, of bones . . . xiv 7

process of reparation of . . vii 450

retentive apparatus in . . xiv 7

splinters in . . . . . xiv 10

starched bandage for . . xiv 11

ununited, cases of . . . ix 269

cured by seton . . xii 255

silver wire in . . . ix 269

treatment of . . . xiv 11

Fractures, and dislocations, Sir A. Cooper on . . . x 103, xiv 28

atrophy of bone in . . . iv 153

Beaumont's apparatus for . vi 313

Chelius on treatment of . . xx 118

compound, amputation in . xiii 172

Dr. Walker on . . xxiii 256

compression in . . . vi 316

consolidation of . . . v 248

diagnosis of . . . . xx 117

Dieffenbach's apparatus for . vi 313

disunion of, from paralysis . iv 152

disunited, Mr. Liston on . . v 460

experiments on, in Berlin . vi 313

Froriep on treatment of . . vi 313

immoveable, apparatus for . vi 310

in children, treatment of . xii 544

in drowning, Mr. Taylor on . xxi 485

in the aged, Chelius on . . xx 117

Fractures, Lecat's opinion on, in 1734 vi 312

M. Larrey's apparatus for . vi 313

treatment of . . . vi 312

M. Moscati's treatment of . vi 312

M. Munaret's gutter for . . vi 319

moulding tablets for . . vii 583

Mr. Amesbury's apparatus for . vi 311, 318

M. Sautier's treatment of . vi 319

M. Velpeau on treatment of . vi 315

observations on . . . xxi 143

of long and flat bones, union of xi 463

long bones, Mr. Liston on . xxii 369

neck of femur, new bed for . xvi 486

radius, Lisfranc on . . xiv 6

thigh, new mode of treating xvi 508

old, tenotomy in . . . xiii 239

process of reunion in . . iv 368

purgatives inapplicable to . xiv 11

recoveries from, in the old . xiv 9

starch first used for . . vi 314

starched bandaging in . . vi 314

statistics of, in America . . vi 258

in Calcutta . . viii 592

in London . . vi 310

treatment of . . . . . vi 308

by Assalini . . vi 312

by the Moors . vi 313

by the Swiss . vi 312

in Brazil . . vi 312

in Corsica . . vi 312

in Greece . . vi 312

in Italy . . vi 312

in Spain . . vi 312

treatments of, compared . . vi 318

Turkish apparatus for . . vi 311

un-united, Sir B. Brodie on . xxii 166

use of iodine in . . viii 523

use of white of egg in . vi 312, 319

vicious consolidation of . . vi 309

Fraenkel, M., on the teeth . . viii 158

Franc, Prof., on contractions of urethra . . . xiii 312, 327

France, and her peasantry . . xiv 455

climates of . . . . xii 169

foundling hospitals in . . xiii 289

Italy, and Germany, memorandum on xiv 309

medical profession in, division of i 495

medico-legal proceedings in . ii 392

registration of infants in . . i 333

France, Mr., on crystalline lens . xxiii 425

on the pathology of iritis . xxiii 418

Francis, trial of, remarks on . . xvi 85

Francis, Dr., on sudden death from disease . . . . . xxiii 424

Franecker, college of . . . vi 280

Frank, Joseph, his Prax. Medic. . xviii 310

Franklin, Dr., on water-drinking . xxiv 520

Frankum, Mr., gossiping, trashy work of xiv 215

on pendulous abdomen . . xiv 215

ridiculous pathology of . . xiv 215

Franz, Dr., his cure of blindness . xii 567

on preserving the eye . . x 39

Franzensbad, spa of Egra . . xiv 344

Freckle, the summer, cause of . xxiv 70

Freckles, the dermis the seat of . vii 239

FREDERICK WILLIAM'S medico-chirurgical institution in Prussia i 297

Free living, its effects on the stomach vi 177

FREEMAN, case of poisoning by . xx 334

FREEMAN, Dr., his mode of hydropathy xvi 471

Freezing, Hunter's experiment on . v 50

speculation on . iv 82

revival after . . . . . xv 331

Frémissement cataire, of Laennec . i 448

Frenchified nomenclature, remarks on iv 195

French army, in Egypt, mortality in vi 154

hospitals, house pupils in . i 494

hospital surgeons, blunders of . xi 219

medical institutions . . i 494

journals . . . i 223, 547

officers, instructions to . xxiii 80

practice, state of . . i 495

military, medical, memoirs . xxiii 74

*savans*, blindness of . . xxiv 400

school of medicine . . iii 384

therapeutics, account of . . iii 449

FRÈRE COME, lithotomy by . . xii 392

FRÈRE JACQUES, lithotomy by . xii 392

FRESENIUS, Dr., on chemical analysis xvii 244

Fresh-meat rations, benefit of . . viii 222

FRICKE, Dr., his treatment of syphilis xv 468

on compression in inflamed testicle ii 253

on episioraphy . . . vii 562

on varicocele . . . . . iv 232

Friction, and shampooing, in sprains iv 232

Dr. Kilgour on the use of . i 357

on the back, benefit of . . v 146

-sound . . . . . . . ix 314-16

-sound, in pericarditis . . xxiv 549

Friendly societies, observations on . xxi 16

sickness as to . . xxi 15

Fright, apoplexy from . . . xi 235

FRISCH, on nux vomica in diarrhœa i 256

Frog, cerebral nerves of . . vii 237

effect of vinegar on skin of . vi 415

galvanoscopic . . . . xxiii 396

nerves of . . . . . vii 541

peculiar electric current of . xxiii 400

experiments on nervous system of xi 302

Frontal bone, case of caries of . . xxi 471

sinus, case of fungus of . . xxi 186

FRORIEP, Dr., eulogy of . . iv 168

his surgical copper-plates . iv 168

Frost-bite, amputation from, cases of vi 550

effects of . . . . xviii 218

gangrene from, amputation in xi 320

of foot, remark on . ii 29

refrigerants in . . xi 317

Fruits, and farinacea, the food of man xxi 148

composition of . . . xvii 20

fleshy, as articles of diet . . xvii 20

Frustulia salina, oxygen from . . xxiii 494

Frying of food, remarks on . . xvii 37

FUCHS, Dr., on health of professions x 373

on softening of brain . . xi 464

Fucus esculentus, as a bougie . . ix 222

Fuligokali ointment for psoriasis . xv 395

Fuligo splendens, against abortion . xxi 472

Fumigating powder of iodine, &c. . xxiii 371

Fumigations in hooping-cough . i 574

of cinnabar, new method of . ii 555

Functional and structural analogy . vii 177

Function, alteration of, in the body xxiii 51

and use of an organ . . xx 165

unity of . . . . iv 532, vii 178

Functions, animal, classes of . . v 490

division of . . xiii 476

antagonism between . . vii 179

balance of, disputed . . xvi 441

mental, remarks on . . xiii 477

of animal life . . . xiii 476

of body, general view of . . xiii 475

of nervous system . . . xiii 477

organic, of vegetables . . xiii 476

revolutions of, cures by . . xxiii 586

Fundus uteri, in feet-presentations . iii 417

Funeral societies, bad effects of . xvii 405

Fungiæ meandrinæ, nutrition of . xix 556

Fungi, in mouth and intestinal canal xxiii 509

in porrigo lupinosa . . xxiii 509

production of, in plants . . iv 20

used as food . . . . . xvii 21

Fungin, nature of . . . . . xvii 21

vegetable principle of . . iv 9

Fungoid disease, Dr. Baring on its nature i 473

of stomach, case of . xxiv 142

testicle, diagnosis of . . i 474

Fungous and scirrhous testicle, diagnosis of i 474

Fungous diathesis, M. Bonnet on . xxii 63

disease, rarely if ever local . i 477

testicle, and hæmatocele, diagnosis of i 476

aspect of, patience important in its diagnosis . i 475

castration for, when proper and not i 477-8

duration of . . . i 472

hæmatocele mistaken for . i 476

hard at first . . . i 475

inguinal glands in . . i 473

ulceration of skin rare in . i 472

Fungus genu, of German authors . x 335

hæmatodes, French misled by term i 470

hæmatodes of eye, extirpation of xviii 420

of bladder . . . xi 361

medullary, Dr. Baring on . i 469

of dura mater, case of . . vi 541

of frontal sinus, case of . . xxi 186

of joints . . . xxi 126, 128

of testicle cured by castration . i 477

Dr. Baring on . . . i 469

etiology of . . . i 476

German authors on . . i 471

primary seat of . . i 472

Funic bellows-sound . . . x 274

Funis, cut or tied, in privies . . vii 138

desiccation of, in infants . xi 200

distended, on emptying . . xiv 369

Dr. Kennedy on sounds of the i 94

evidence of life from state of . xi 200

Funis, knot on, in dead fœtus . . xiii 568
length and twisting of . . ii 360
Michaelis on reposition of . iii 523
pressure on, cause of death from xv 318
procidentia of, treatment of . xix 417
prolapsus of . . . vii 291, xv 314
Dr. Collins on . . ii 90
Dr. Denman on . . ii 91
Dr. Michaelis on . . ii 90
in 16,434 labours . . ii 73
management of . . xiv 559
treatment of . . . xv 319
round neck of child, query on ii 75
remarks on shortening of . ii 359
short, cases of . . xiv 582, 583
shortness of . . . . xii 480
strangulation of infants by . ii 415
treatment of in West Indies . ix 532
Funis umbilicalis, reposition of . i 588
Furnas, in the Azores, hot spring at xiv 355
St. Michael's mineral waters of xii 224
FURNIVALL, Dr., on consumption vii 536, xvii 531
Furor, maniacal, or acute mania . xx 19

GAILLARD, the Abbé, on foundlings xiii 289, 293
Galactorrhœa, remarkable case of xx 538
GALEN and HARVEY, remarks on xv 449-50
GALEN, a dexterous anatomist . vi 385
analysis of his works . . vi 385
an anecdote from . . . vi 385
his disquisition on the eye . xv 453
doctrines of physiology . i 381
knowledge of arteries . vi 385-6
of the nerves . vi 386
opinions on nutrition . vi 385-6
on respiration . vi 386
historical account of . . xxiii 94
his views on the nervous system ix 108
ignorant of the circulation . vi 386
on generation . . . xv 453
on final causes . . . xv 441
on formation of the fœtus . xv 454
on nerves of sensation and motion i 382
on sensation and motion . vi 387
on spinal marrow and nerves . vi 387
on the catamenial discharge . vi 387
on the hands and feet . . xv 444
on the heart . . . . xv 448-50
physiological reasoning of . vi 386
the works of, Dr. Kidd on . ii 603
Galeopsis glandiflora, preparation of xx 449
GALL, his doctrine of the cerebellum xxii 534
his first idea of phrenology . ix 196
his views on nervous functions ix 116
on phrenology . . . ix 190
Gall-bladder and ducts, inflammation of xxi 51
enormously distended xvi 320, xx 222
fibres in the walls of . xi 524
mistaken for hepatic abscess ix 482
morbid anatomy of . . xv 99
Galley-worm, structure of . . xx 489
Galls, use of in chronic dysentery . iii 145
Gall-stone, Dr. Seymour on . . xxiv 142
Gall-stones, diagnosis of, from jaundice xxii 317
Dr. Budd on . . . xxi 58
Durande's remedy for . xiii 562
observations on . . xiii 561
treatment in . . xiii 562, xxi 59
Galvanascope from frog's leg . xxiii 396
Galvanic obstetric forceps . . viii 566
puncture, effects of . . v 35
Galvanism as a remedy, remarks on ii 147
by needles in aneurism . . xxiii 407
Dr. Philip on . . . ii 147
effect of, in digestion . . vi 527
on uterine action . . xxiii 279
for deafness, remarks on . iii 84
function of stomach restored by ii 137
in diseases of the eye . xii 542, xiii 242
in suspended animation . . x 584
paralysis cured by . . . xi 524
the cause of secretion, &c.? . ii 136
Galveston, city of, in Texas . . xii 434
GAMA, M., and the King of Sweden i 305
Gambia, and Sierra Leone, fever at xi 368
dreadful insalubrity of . . xi 367
St. Mary's on the . . . xi 366
summary of mortality at . xi 368
temperature of . . . xi 367
Gamboge, chemical characteristics of xiii 63
observations on . . . xiii 63
Game, putrid, on the noxious effects of xxiii 161
Ganglial cavities, pathology of . xviii 83
Ganglia, bursting of, by blows . xxi 292
cephalic, their functions . xxii 495
formation of nervous . . xi 299
functions of the, Unzer on . xxiv 185
hemispherical, Mr. Solly on . xxiv 573
in fishes, Mr. Owen on . v 494
in typhus, state of . . . xv 97
investigations of . . . v 300
nervous, discovery of new . xv 232
structure of . . . xiv 283
of the spinal nerves . vii 543, 544
sensitiveness of the . . xvi 160
sensory, and cerebrum, on . xxii 504
sympathetic, office of . . xi 298, 304
Ganglionic, remarks on the term ii 135
centres, in animals . . viii 511
globules . . . . . vi 412
nerves, functions of . . iv 476
influence of . . . v 336
system, effects of shock on . xiv 57
Dr. Philip on . . . ii 133
minute anatomy of . . xviii 142
use of, to the heart . . iii 11
Ganglion, median of the remora . xxii 498
patellæ, or housemaid's knee . iii 151
spinal, structure of a . . xxiv 130
the geniculate, nature of . xxii 281
the proper restiform . . xxiv 575
Ganglions and bunions, Mr. Key on iii 151
Ganglions, intimate structure of . vi 404, 412
nervous, act as little brains . ii 133
structure and functions of ii 133

Ganglions, of nerves, structure of . vii 502

treated by puncture . . iii 151

use of acupuncture in . . xxii 54

iodine in . . . viii 523

Gangræna senilis, a faulty term . xiv 85

on the cause of . vi 157

commencement of . vi 157

treatise on . . xiv 84

treatment of . . xix 528

urodialytica . . xvii 118

Gangrene, after ligature for aneurism xxii 110

and ulceration, Dr. Lebert on xxii 25

boundary line of . . . ii 24

comparative danger in . . ii 29

congenital, case of . . . xx 549

definitions of the term . . xi 317

dry, amputation in, case of . xiv 250

arteritis in . . . xxiv 412

case of, in a child . . ix 442

in arm, case of . . xiv 250

erroneous ideas prevalent on . ii 29

from diseases of the heart . ii 333

ergot, query on . . xvii 501

obstruction, age in . . xiv 86

bruits in . . . xiv 87

causes of . . xiv 86

chronic . . . xiv 88

diagnosis of . . xiv 89

duration of . . xiv 89

heat in . . . xiv 87

limits of . . . xiv 88

nature of. . . xiv 85

phlyctenæ in . . xiv 87

prognosis of . . xiv 89

sex in . . . xiv 86

sites of . . . xiv 86

stages of . . . xiv 87

treatise on . . xiv 84

treatment of . . xiv 90

from starched bandage . . viii 558

hospital, remarks on . . xi 320

in children, report on . . xvii 564

infantile, causes of . . . vii 476

Dr. Richter on . . vii 470

treatment of . . . vii 476

varieties of . . vii 470, 476

M. Josse on amputation in . iii 123

Mr. Travers on amputation in. ii 28

observations on . . . xviii 113

of extremities, in Spain . . vi 450

genitals, in female children . xxi 470

in females . . . vii 473

hand, case of . . . vi 66

heart, instance of . . ix 434

lungs . ii 547-8, xi 408, xx 226

in the insane . . . ii 546

mouth, in children . . xxiv 46

actual cautery in . . x 585

of infants . iii 444, vii 471, xi 203

nates, generally fatal . . ii 29

penis, seldom fatal . . ii 29

serous membranes . . iv 52

skin, infantile . . . vii 474

Gangrene of skin, in a child . . xxiii 308

uterus . . . . vi 539

senile, case of . . . iii 122

M. Josse on . . . iii 121

remark on . . . xxii 172

use of cold water in . iii 121, 123

spontaneous, of the surface . xvii 565

spreading, on operating in . xiii 567

Gangrenous inflammation—

characteristics of . . ii 28

distinguished from gangrene ii 28

fever in . . . . ii 28

Mr. Travers on . . ii 28

opium in . . . ii 30

pulse in . . . . ii 29

treatment of . . . ii 30

Gardner, Mr., his Great Physician xv 228

on kephalosis . . . vii 222

Gargouillement, an important sign i 213

Garlic injected into the veins of a dog i 245

Garotillo of the Spaniards . . xv 326

Gaseous contents of the blood . xxi 544

theory of disease . . . xxi 146

Gas, absorption of, experiments on xxiii 142

chlorine, cure of hydrocele by ii 267

explosions of, from vagina . xviii 21

in corpses, sometimes moves them ii 412

in the circulation, death from . vii 249

Gases, absorption of, from stomach xiv 307

contained in the blood . . xxi 146

development of, in the body . xxii 324

from putrifying matter . . xi 447

Magendie on the study of . viii 322

mechanical properties of . xix 8

of the intestinal canal . . xvii 436

permeability of textures to . viii 322

Gaspard's experiment on poisons i 559

Gasserian ganglion, connexions of . xiv 382

Gastein, hot water of . . . xiv 352

Gastralgia, and gastritis, diagnosis of xviii 210-1

M. Barras on . . xviii 208

hypochondria in . . . xviii 211

symptoms of . . . xviii 211

Gastric acid, softening of stomach by i 138

and cardiac disease, case of . vii 127

digestion . . . . . xix 269

fluid, Mr. Paget's report on . xxi 560

properties of . . . xix 270

secretion of . . . xix 269

fever, sources of (App. to vol.) xi 35

juice, absent in fasting . . vi 176

acid of . . . . xvii 434

action of . . . xiv 512

and germ, similarity of . xv 146

as seen during life . . vi 175

diffusible in water . . v 401

Dr. Prout on . . . xi 332

examination of . . vi 177

experiments on . . vi 172

extracted during life . vi 177

hydrochloric acid in xvi 148, xxiv 273

its digestive powers . vi 178

L'Heritier on . . . xvii 434

Gastric juice, Liebig on . xiv 512, xxiv 272

limits of its secretion . vi 178

no such thing as acid . xvi 222

not the cause of hunger . ii 136

restrained by irritation . vi 78

by passions . . vi 78

secretion of, denied . xvi 222

visible secretion of . . ii 376

papillæ, description of . . vi 175

ramollissement in children . xvii 560

secretion, strange experiments on xix 270

with hepatic disease . . vii 126

Gastritis, and brain affection, diagnosis of xix 26

chronic, anorexia in . . vii 124

cough in . . . vii 124

dyspepsia from . . ix 477

Dr. Stokes on . . ix 477

in children . . . vii 125

nausea in . . . vii 124

pains of chest in . . vii 124

percussion in . . . vii 124

quick breathing in . . vii 124

tongue in . . . vii 124

vomiting in . . . vii 125

Dr. Stokes and others on . ix 474

four varieties of . . . xv 93

in children, report on . . xvii 560

internal use of ice in . . ix 476

milk diet in . . . . xxiv 47

morbid appearances in . . xi 415

submucous, anatomy of . . xv 93

treatment of . . . . ix 476

Gastrobrosis in young women . xx 315

Gastro-cephalitis, quinine in . . viii 61

treatment of . . . viii 59

-duodenitis, affecting liver . xii 81

Gastrodynia, and gastritis, diagnosis of xxii 297

and pyrosis, treatment of . x 43

diet in . . . . . x 43

hydrocyanic acid in . . viii 358

Mr. Pereira on . . . viii 357

use of milk in . . . xix 29

Gastro-enterite, mercurial inunction in viii 420

M. Louis' work on . . vi 41

-hepato-peritonitis . . xii 79

Gastroperiodynia, Dr. Wise on . iii 215

in India . . . . . iii 215

Gastrorrhea, follicular, remarks on xii 505

Gastrotomy, for diseased ovaria . xvi 387

in disease of cæcum . . ix 250

in extra-uterine pregnancy . xiii 545

in the cow . . . . xii 253

report on . . . . . xvii 548

Gauthier, Dr., on iodine in syphilis xx 482

Gavarret, M., on medical statistics xii 1

Gavin, Dr., on feigned diseases . xviii 117

Gay, M., on apoplexy . . . xvi 272

Gazette Médicale de Paris . . i 225

Geary, Dr., on fever . . . v 336

Geddes, Dr., on diseases of India . xxiii 106

Geddes, Mr., his letter to the editors iv 279

Geddings, Dr., his ointment for piles i 579

on adipose tissue . . . i 153

Geddings, Dr., on bloody tumours of the scalp . . . i 182

diseases of the aorta . i 157

Geese, livers of, how enlarged . i 346

Geigel, Dr., on the origin of disease xxi 145

Gelatine, and horn, formation of . xiv 513

extracted by acids, as food . xiii 496

formula for . . . . xix 250

French commission on . xiii 486, 495

nutrition by . . . . xiv 510

nutritive qualities of . xiii 253, 495

oxidation of . . . . xii 532

use of, as food . . . xix 563

Gelatinous principle of aliment . xvii 11

tissues, metamorphosis of . xv 506

Gellerstedt, Dr., on tubercular phthisis . . . . . xxiii 429

Gely, Dr., on suture of the intestines xxiii 1, 24

Gemignani, Dr., on the neuroses . xix 555

Gemmiparous propagation in polypes vii 184

Gendrin, Dr., his originality . x 74

his stilted style . . . x 79

his treatise on medicine . . x 55

General anatomy, initiated by Bonn i 388

its effect on physiology . i 388

Generalization, and facts, remarks on v 318

false, example of . . . v 337

in reference to God . . v 342

physiological . . . v 338

Generalizations and laws, distinction of xx 142

General practitioners, association of xix 614

Generation, ancient physiology of . xv 453

equivocal, remarks on . . i 392, iv 28

fissiparous . . . . vii 184

in animals . . . . vii 184

physiology of . . . xi 303

report on . . . . xvii 270

spontaneous . . . . xi 398

Generations, alternation of . . xxiv 439

Genesis, observations on the Book of xxiv 480

Geneva college, New York . . ii 592

Geneva, hospital for incurables at, bad i 498

mean age of 6338 males in . i 194

Medical Society of . . xxi 274

mortuary registers of . . i 194

population of . . . xv 339

suicide in . . . . ii 277

Geniculate ganglion, nature of the . xxii 281

Genital organs, anatomy of . . xix 586

cancer of . . . xxii 306

tumours adjacent to . xviii 34

system, motions of . . . xi 296

Genitals, elephantiasis of, in China xxii 187

female, gangrene of . . vii 473

gangrene of, in female children xxi 470

male, mortification of . . ix 571

report on the nerves of . xxii 288

Genito-urinary epithelium, scaling of viii 131

organs, diseases of the . v 467

Genius, observations on . . xxiv 217

the school, future failure of . ii 387

Gentili da Fuligno, history of . xxi 279

Gentleman, medical, the genuine . xxii 161

Gentlemen, comparative age at death of xx 427

Genus and species, on the terms . iii 371

GEOFFRY, M., his case of poisoning by arsenic . . . . i 572

Geological causes of fever . . xx 211

organic remains, Dr. Clark on . i 392

relations of phthisis and typhus xxii 211

Geology, Dr. Lee's Elements of . xi 224

Professor Ansted's . . . xix 554

utility of the study of . . ix 425

Geophilidæ, development in . . xx 491

GEORGE, Mr., on the claims of Prochaska xxiii 215

GEORGET, M., and animal magnetism vii 322

his dying declaration . . iv 447

Georgia (U.S.), Medical College of ii 591

GERBER, his General Anatomy . xiv 478-9

GERDY, M., his operation for hernia vi 348

on morbid anatomy of bone . ii 535

on treatment of hernia . . ii 566

GERHARD, Dr., on typhus fever xii 293, 306

Germ, and gastric juice, similarity of xv 146

original matter of . . . ix 4

-vesicle of the ovum . . ix 6

German, ancient, respect for women v 444

German character, singular feature in iv 121

hematophobia, exposition of . xvi 475

medical institutions . . i 499

journals i 217, 545, ii 232, iii 460

medicine, on mysticism in . iii 450

physicians, account of . . xxiii 545

physiologists, observations on . xvi 172

school of physiology . . v 82

writers, characteristics of . i 469

industry and patience of . i 469

Germany, advancement of medicine in iii 452

its effects on imagination . iv 122

late scientific discoveries in . iii 582

medical sects of . . . xvi 472

state of homœopathy in . xxii 565

Germinal membrane . . . xx 290

in the embryo . . xv 266

vesicle, and spot . . . xvi 517

changes in the . . xv 263

of plants . . . iv 8

GEROMINI on Dropsy, translation of iii 438

Gestation, Dr. Berthold on . . xxii 291

extra-uterine . . . x 247

of cows, Earl Spencer on . xi 273

remarks on . . . ii 404

influence of, on the breast . x 123

its connexion with menstruation iv 464

period of . . . . . xix 587

human . . . iv 463, vii 136

in French law . . ii 403

statistics of . . . iv 465

protraction of . . . iv 464

until 310th day, case of . ii 404

GHINOZZI, Dr., on clinical medicine xxiv 360

GIACOMINI, Prof., on clinical medicine xxiv 360

GIACOMINI and RASORI, doctrines of xxi 159

Giant O'Brien and John Hunter . iv 83

Giants and dwarfs, remarks on . viii 18

GIBBES, Sir G. S., on diseases . xxiii 501

Gibraltar, diseases of the lungs in . viii 220

geological formation of . . ix 72

yellow fever of . . ix 71, 73, x 494

Gibraltar-fever, causes of death in . x 500

diagnosis of . . x 505

French commission on . x 495

identity of, &c. . . x 504

morbid anatomy of . x 496

mortality of . . x 503

not fatal, symptoms in . x 502

rarity of second attacks of x 505

sporadic . . . x 508

state of liver in . . x 500

symptoms in fatal . x 501

symptoms in mild . x 503

treatment of . . . x 506

GIBSON, Dr., his rambles in Europe xii 232

GIBERT, M., on diseases of the skin xx 521

on tinea . . . . . xiii 233

Giessen, tribunal at, Mulder on . xxiv 273

GILBERT, Mr., flattering compliments to xiv 536

on pulmonary consumption . xiv 536

GILCHRIST, Dr., on treatment of wounds xxii 238

Gilders' malady, effect of civilization on xviii 250

Gilding surgical instruments . . xiv 233

GILES, Mr., on urinary malformation xx 511

GILKREST, Dr., on yellow fever . iv 166

Gin-liver, the disease so called . ii 463

GINTRAC, M., on intra-thoracic tumours xxii 216

on nervous irritability . . xxi 417

GIOJA, on vital statistics . . iii 41

Girls, tight-lacing of, mischief of . xi 426

GIROLAMI, on Gentili da Fuligno . xxi 279

Gladiator, the dying, John Bell on . xiv 528

GLAISHER, Mr., his hygrometrical tables xxiv 283

Glandered horses, law in Berlin on . vi 116

Glanders, acute, in man, cases of . xiii 538

symptoms of xiii 32

in the horse . . xiii 30

after farcy, fatal case of . . viii 552

and farcy, Mr. Percival on . xxii 35

artificially produced . . xi 230

case of fever from . . . xxiii 507

causes of . . . . . xxii 40

chronic, in man, history of . xiii 34

in the horse . . . xiii 29

contagiousness of . . . xxii 41

contagious properties of . . xiii 37

diagnosis of . . . xiii 36, xxii 38

gangrenous acute . . . xiii 31

in diagnosis of typhus . . xxiii 507

injection of creosote in . . ii 170

in man, and horse, identity of . xiii 38

in man . . . vi 113, xiii 31, 39

by infection . . . vi 115-16

by inoculation . vi 115-16, 120

cases of . ii 241, xiv 248, xviii 335

case of, by infection . vi 117

chronic form of . . vi 121

description of . . . vi 117

diagnosis of . . . vi 118

Dr. Elliotson on . . vi 115

from inoculation . . . xiii 33

Glanders, in man, history of . . vi 113

mortality of . . vi 118

mortrimory of . . vi 118

Mr. Travers on . vi 114

non-eruptive form of vi 120

three varieties of . vi 115

treatment of . . vi 119

violent case of . . iv 255

in the horse, history of . . xiii 29

typhus from . ii 243

mode of transmission of, to man xiii 40

origin of . . . . . xxii 43

post-mortem appearances in . xiii 33

pustular acute . . . xiii 30

Schneiderian membrane in . xiii 30

seat and nature of . . . . xxii 45

symptoms of . . . . . xxii 37

transmission of, from man . xiii 235

treatment of, ineffectual . . xiii 40

varieties of . . . . . xxii 39

Gland-cells, secernent, development of xix 565

Gland, thymus, comparative anatomy of xx 162

treatise on . . . xx 159

in the tip of the tongue . . xxi 281

morphological element of a . xiv 292

submaxillary, extirpation of . xiii 238

Glands, absorbent, structure of . xiv 290

aggregated, structure of . . xiv 292

closed, tabular, structure of . xiv 292

Cowper's, in the female . . xvi 155

dropsy of the ducts of . . v 181

ducts of, structure of . . xiv 293

Duverney's, description of . xvi 156

enlarged, treatment of . . x 54

functions of . . . . . xiv 292

inguinal, in fungous testicle . i 473

intestinal, anatomy of . . vi 218

Dr. Albers on . . . v 184

minute structure of . . i 521

*plate* of . . . . i 528

structure of . . . i 236

labial, M. Sebastian on . . xv 227

lymphatic, history of . . iv 333

inflammation of . . ii 558

structure of . . . xx 297

nucleated cells the elements of ix 523

of Brunner, anatomy of . . i 527

diseases of . . xi 420

of large intestines, two kinds of i 527

Lieberkuhn, in intestines . i 526

Peyer, corpuscles of . . i 523

diseases of . . xi 420

general anatomy of . i 522

in man . . . i 524

structure of . i 236, xiv 291

stomach, action of . . xxiv 278

structure and action of . . xxi 569

reticulated, structure of . . xiv 293

salivary, extirpation of, results of xiv 221

sebaceous, of the skin . xv 380, xxi 200

secernent, structure of . . xiv 291

secreting, Müller on . . ix 533

Glands, solitary, of the colon . . xxiv 335

sudorific, account of . ii 438, vi 514

sudoriparous, of the skin . . xv 380

varieties of, in the larynx . xxiv 123

vascular, report on . . xix 570

structure of . . . xiv 294

works on . . . xiv 294

vesicular, action of . . . xiv 293

without ducts, report on . xxi 566, 569

Glandulæ simplices, minores et majores i 527

solitariæ, of intestines . . i 527

system, predisposing to disease xx 344

Glans penis, veins of . . . xix 509

Glasgow bridewell, no insanity in . xii 502

fever-hospital, table of cases in . . (App. to vol.) xi 30

infirmary, report of . . v 607

Glasgow, description of the wynds in xiv 456

drunkenness in . . . xiv 456

great unhealthiness of . . xxi 13

increase of fever in . . xi 29

intemperance and mortality in . xxiv 518

medical topography of (App. to vol.) xi 44

report on food for the poor . xiii 486

sad state of the poor in . . xi 26

sanitary report of . . . xviii 492

typhus fever in . . . xii 294

vital statistics of . . xi 1, xxii 215

Glaucoma, appearances in . . x 24

cataract from . . . xviii 55

cause of . . . . . xviii 52

change in the iris in . . xvi 116

choroiditis the cause of . . xiii 505

concave appearance of . . xviii 53

different stages of . . . xvi 112

diagnosis of . . . . xvi 116

Dr. Mackenzie on . . vi 274, xvi 113

Dr. Sichel on . . . xvi 110

his cases of . . . xvi 116

Mr. Middlemore on . . ii 349

effusion in . . . . . xviii 54

green colour in . . . xviii 54

incurability of . . . xvi 117

nature of . . . ii 269, xvi 113

pathological anatomy of . . xvi 115

change in . . . xvi 115

pathology of . . . . ii 348

senile, Mr. Middlemore on . ii 349

Van der Kolk on . . . xviii 48, 51

*Glaukòs*, meaning of the Greek word xvi 111

Glisson, his Tractatus de Ventriculo v 523

on reflex nervous impressions . v 523

Glisson's capsule, anatomy of . . xx 135

Globule, compound inflammatory . xxii 333

Globules, compound, in inflammation x 259

in blood, proportions of . . xvii 137

in health and disease . . xiv 240

of blood, Magendie on . . viii 351

motion of, in vessels . viii 339

produced from albumen . . xix 168

Globulin of blood . . . . vi 436

Glossanthrax, rapidly fatal . . xiii 42

Glossology, Dr. Ridge's essay on . xvii 524

Glosso-pharyngeal nerve, functions of . v 308, vii 547, viii 265

the nerve of taste . ii 600

Glosso-pharyngeus nerve, functions of xviii 139

and nucleus . xviii 137

Glottis, caustic squeezed into . . vi 165

closure of, in deglutition . xiii 227

effect of section of nerves on . iii 29

feeling the, impossible . . v 434

inflammatory œdema of . . xi 403

œdema of . . . . . v 431-2

diagnosis of . . xviii 296

expiration easy in . xviii 297

M. Valleix on . . xxi 412

nature of . . xiii 537

scarification in . . xviii 298

treatment of . . xviii 297

scalded, case of . . iii 562, vi 270

spasm of . . . . . v 438

Dr. Joy on, referred to . ii 240

Glover, Dr., on scrofula . . xxiii 513

Glover, Mr., on the rete mucosum vi 574

his suture for wounded intestines xxiii 16

Gluge, Dr., on diseased kidney . v 554

on influenza . . . . . vii 95, 103

on softening of the brain . . x 259

Gluten, as food, experiments on . xiii 490

formation of, by vegetables . xiv 494

long of digestion . . . xiii 493

nutrition from . . . xviii 15

nutritive qualities of . xiii 254

of wheat, a compound substance xvii 15

procuring of, from wheat flour . xiii 488

the nutritious substance in vegetables . . . . . xiii 488

Gluten and vitellin . . . xvi 285

Glyptodon, structure of the tooth of xxi 71

Gmelin, and animal magnetism . vii 318

Gobee, Dr., his clinical contributions xv 399-402

Goddard, Dr., his Plates of the nerves iv 497

his translation of Moreau . xix 416

Goître and cretinism, difference of . xvii 514

Goître, at the lake of Como . . viii 113

biniodide of mercury in . . xix 519

calcareous origin of . . viii 115

cause of , . . . . xx 174

cured by minute doses of iodine viii 427

endemic, treatise on . . xx 168

geology in relation to . . viii 108

Indian localities in relation to viii 108, 111

in England, Dr. Inglis on viii 112, 116

in India . . . viii 116, 118

tabular view of . viii 107, 110

in Nepaul, Mr. Bramley on . viii 116

in relation to dolomite . . viii 113

M'Clelland on the cause of . viii 106

not from drinking snow-water . viii 104

origin of . . . . . xii 119

sea-air as preventive of . . viii 115

treatment of . . . . . viii 119

use of iodine in . . . viii 119

(see Bronchocele.)

Golden incision for hernia . . vi 345

Gold, chloride of, formulæ of . . ii 211

formula of, for rheumatism . xiv 418

its use in syphilis . . . v 20

preparations of, in syphilis . xxiii 369

printing in, poisonous . . vii 291

Gondret, Dr., his pommade ammoniacale . . . . vii 65, 67

on derivation . . . vii 56, 73

Gonorrheal conjunctivitis . . x 393

inflammation of the iris . . x 397

ophthalmia . . . . . x 25

puncturing cornea in . x 397

three forms of . . x 394

treatment of . . x 395, xviii 408

orchitis, origin of . . . xvii 77

Gonorrhea and syphilis, identity of x 384, xxiii 347

Gonorrhea, abortive treatment of . xii 370

a cause of rheumatism . . xviii 522

analogy to ophthalmia . . ii 504

cause of chordee in . . xii 427

contagion of . . . . xiii 239

copaiba balsam, by enema, in . i 578

false, or balanitis . . . x 388

if purulent, contagious . . xii 368

in females, lunar caustic in . iv 540

nitras argenti in . iv 258-9

suppository for . xi 527

injection of ioduret of iron in . x 390

of tannin in . . . x 391

injections in, compared . . x 391

inoculation of . . . . x 384

nitrate of silver in . . x 390, xiii 61

non-contagious state of . . xii 368

observations on . . . xi 508

popular cure for, in Paris . xii 50

pure, a specific disease . . xii 363

remarks on cure of . . xiii 313

secondary affections from . xii 368

symptoms of . . viii 426, x 392

syphilis from . . . x 385

simple, and virulent . . xii 364, 367

causes of . . . xii 363-4

cure of . . . . xii 364

not contagious . . xii 363

Sir A. Cooper on mercury in . x 391

swelled testicle from . x 392, xii 371

syphilides from . . . xxiii 363

treatment of . . . . x 390

use of chloride of zinc in . xi 527

leeches in . . . ix 240

Velpeau on remedies for . . xii 459

Good breeding in medical men, on . xxii 123

Goodlad, Mr., misrepresentations of xii 121

on nervous affections . . xii 121-8

Goodsir, Messrs., anatomical and pathological observations, by xx 289

Goodsir, Mr., on fever at Anstruther xii 316

on nutritive absorption . . xv 268

the human teeth . . vi 583

the intestinal villi . . xiv 566

the teeth . . . vii 571, viii 158

GOODSIR, Mr., on vegetable organisms xiv 246
Goose-foot, stinking, an emmenagogue vii 235
Goose-liver, fat, disease in man from xvii 25
GORHAM, Mr., on intussusception . vii 453
on the pulses of infants . . v 268
GORING, Dr., his microscopical illustrations x 550
Gottenburg, scientific meeting at . x 549
Göttingen university, account of . i 500
GOTTSCHE, Dr., on the retina . . iv 499
Gout and hysteria, relations of . xii 73
and rheumatism . . . xx 268
diagnosis of . . xiv 415
Dr. Todd on . xvi 460, 471
identity of . . vi 376
pathology of . . xii 120
Gout, action of colchicum in . . xii 73
a first attack of, described . xxiv 148
a morbific matter the cause of . viii 497
caution on purgatives in . . xvi 464
cautions on treatment of . . xvi 464
colchicum as a preventive of . viii 500
cured by animal electricity . xi 233
cure of, by Vichy waters . . xiv 348
diet in . . . . . . xvi 466
dogs subject to . . . xxiv 88
Dr. Ciani's theory of . . ix 238
Dr. Heidler on cure of . . xiv 334-5
Dr. Holland on nature of . viii 497
Dr. Seymour on . . . xxiv 147
Dr. Todd's pathology of . xvi 461, 464
efficacy of colchicum in . . viii 499
exciting causes of paroxysm of . xvi 466
external use of colchicum in . iv 536
morphia in . iv 536
extracts from Dr. Budd on . xvi 464
fit of, from fumes of cider . xxi 372
from stricture of urethra . . iv 133
gravel, and calculus, Dr. Jones on xv 500
in Philadelphia, remarks on . xxi 371
in relation to lactic acid . . xvi 465
lithic acid . . xvi 465
the kidneys . viii 499
its relation to other disorders . viii 499
mineral waters and baths in . xiv 333
Mr. Frankum's diaphoretic for xiv 216
Mr. Parkin on . . . xii 507
nature and cure of . . . vi 352
of morbid matter of . viii 498
not always hereditary . . viii 498
operation of colchicum in . viii 499
pathology of . . . . . xii 73
preventive treatment of . . xxiv 149
relation of, to scrofula . . viii 242
to the liver . . . viii 499
rheumatic, root of colchicum in vi 556
Sir C. Scudamore on . . vii 534
spinal disease from . . xvi 256
symptoms of internal . . xiv 335
the magnet a cure for . . ii 246
therapeutic precept on . . xvi 465
the urine in . . . . . xii 73
treatment of . . . xvi 466, xxi 217
of a paroxysm of . . xxiv 148
Gout, use of ballota lanata in . i 566
benzoic acid in . . xii 398
carbonic acid gas in . xii 509
castor-oil frictions in . iv 243
colchicum in . . . xvi 466
diluents in . . . xvi 466
lactic acid in . . . ix 239
subacid fruits in . . xi 353
Gouty affection of the spinal cord . ix 493
anomalous ailments . . xiv 413
concretions, Dr. Ure on . xiv 52
diathesis in hysteria . . xii 69
new theory on . . xvi 227
new treatment of . . xiii 264
diseases of old age . . xvii 114
finger, curious treatment of a . vi 516
person, peculiar secretion in a xiv 227
renal inflammation . . x 303
subjects, on biliary affections in xii 82
swellings, use of iodine in . viii 523
Governesses, their unhappy condition i 339
Government of the profession xix 217, 551
Governments, on misery caused by xiv 452
GOYRAUD, Dr., on fracture of the radius ii 256
on retraction of the fingers . i 269
Graafian follicles, and contents of . xvi 513
vesicle, account of . . i 610
contents of mature . ix 13
dehiscence of . . . ix 14
first formation of . . xvi 518
in still-born child . . xix 86
M. Baer on the . i 239, iv 461
vesicles, changes in . . x 592
time of bursting of . xvi 524
Graduates, foreign . . . xix 226
Graduations at Edinburgh, 1837 . iv 563
medical, in Prussia . . i 296
Graeffenberg, and houses of Priessnitz xiv 429
Dr. Graham's account of . xxii 428
mode of practice at . . xxii 437
GRAETZER, Dr., on diseases of the fœtus . . . . . xi 212, 216
GRAEFE, his rhinoplastic operation vii 391
Grafting, observations on . . iv 17
GRAHAM, Dr., on the water-cure . xxii 428
GRAHAM, Prof., his Elements of Chemistry . . . . xi 129, 150
GRAHAM, Sir James, new medical bill xix 193
GRAHAM'S staff of life . . . v 411
Grammar, importance of . . xxiv 218
on educating idiots in . . xxiv 11
GRAINGER, Mr., dissent from his theory v 538
his experiments on salamanders v 514
objections to experiments of . v 512
on the spinal cord . . . v 486
GRANTHAM, Mr., his Facts in Medicine xix 247
GRANT and HALL, Drs., v. Mr. Newport v 620
GRANT, Dr. K., his Hooper's Dictionary vii 526
GRANT, Dr. R., on comparative anatomy xiii 217
Dr. Marx on . . . xv 24
Granular disease of kidneys . . x 301
Granulation, healing by . . xviii 280
Granulations from endocarditis . ii 325

Granulations, Dr. Macartney on . vii 439
globular, in the heart . . ii 325
healing of wounds by . . xviii 75
warty, in the heart . . ii 326
weak, cause of . . . . v 196
GRANVILLE, Dr., formation of his lotion vi 504
hallucination of . . . vii 68
his antidynous lotions . . vii 65
aristocratic cases . . vi 503
book tantalizing . . vi 502
character as a writer . . vii 73
excuse for secresy . . vi 504
lotions . . . . . vii 292
own case . . . . vii 72
secret embrocation . . vi 500
six conclusions . . vi 499
hopes to the world from . vi 500
improved stethoscope by . vi 581
on counter-irritation . vi 498, vii 56, 73
on the Spas of England . xiv 309, 318
Germany . xiv 309, 317
painful regret for . . . vi 504
remarks on his secresy . . vi 498
rivalled in newspapers . . vi 503
ruin of the profession by . vi 502
shock from his lotion . . vii 58
strictures on . . . . vii 68
"Granville's Lotion," mode of applying . . . . . vi 502
the only mart for . . vi 504
Grape-cure in Germany . . xix 241
Grasshopper fed with its own stomach ii 136
GRASSI, Dr., on destroying the plague xix 47
on the plague . . . . xix 41
Grass oil of Nemaur, account of . xvi 357
Grate, cottage, remarks on . . xix 16
Grates and stoves, remarks on . xix 16
GRAVAGNA, Dr., on cases of plague xvi 290, 293
Gravel, calculus, and gout, Dr. Jones on xv 500
and stone, new remedy for . xiii 264
pathology of . . . . . xii 73
GRAVES, Dr., his clinical lectures . ix 461, 494
his clinical medicine . . xvi 232
on abscesses of the lungs . xiii 557
cardiac affections . . xiv 241
spasmodic asthma . . xi 264
the pupil in fever . . vi 554
treatment of cholera . v 280
remarks on his writings . . xviii 56
sketch of . . . . . xii 233
Graves, common, at Naples, &c. . xv 522
disgusting scenes in, in London xv 514, 518
on the depth of . . . xv 520
time for reopening . xv 518
regulations on the size of . xv 521
remarks on common . . xv 522
Grave-yards, gatherings from . . x 212
Gravidine in urine of pregnant women xiii 571
Gravid uterus, Dr. W. Hunter on . xvii 530
sounds of circulation in . i 90
GRAVINA, Dr., on ergot of rye . x 554
Gravity, influence of, on the circulation iv 246
its effect on diseases . . iv 246
Gray granulation, Dr. Gross on . xxiii 69
granulations in the blood . vi 135
in the lungs . . . xvi 426
tuberculous . . . xxii 28
nervous matter, properties of . v 492, 593
Grease, from the horse, inoculation with vi 481, 485
history of the . . . xiii 48
in horse, cowpox not from . ix 79
experiments with . . ii 251
remarks on . . vii 296, ix 78
origin of cowpox from . . xiii 48
the form of cowpox in the horse vi 480
produces only cowpox . i 376
Great Britain, on colonization by . xiv 454
public hygiene of . . xviii 492
GREATREX, Mr., on popliteal aneurism xxiii 410
GREAVES, Mr., on mechanism of the heart ii 599
GRECO, Dr., on insanity at Palermo vii 1, 51
Greek philosophic schools, remarks on v 321
physicians, Indian drugs of . xxiii 529
Greeks, medical history of the . xxiii 89
sources of medical science of the xvii 456
Green colour behind pupil, remarks on ii 268
confectionary, poisoning by . xviii 551
evacuations in children . . xxiii 305
fecula in extracts of solaneæ . iii 229
ground in Portugal street, London x 214
GREENE, Dr. G., on the diagnosis of aneurism . . . . . iii 551
GREEN, Dr. H., on diseases of the air-passages . . . xxiv 482
GREEN, Dr. J., on diseases of the skin iv 175
shameless piracy by . . xxiii 372
GREEN, Dr. P. H., on cerebral tubercles vii 576
on infantile mortality . . vii 293
nervous tremor . . vii 583
the seat of tubercle . . xx 97
tubercles in the brain . ix 745, xv 152
GREEN, Mr. J. H., on mental dynamics xxiv 216
on vital dynamics . . . x 545
GREENHILL, Dr., his edit. of Sydenham xviii 527
his Theophilus . . . xv 439
GREENHOW, Dr., on burns . . vii 287
GREENHOW, Mr., case of diseased ovary by . . . . . xx 90
new fracture-bed of . . vi 582
on neuralgia . . . vi 573
the case of Miss H— M—. xix 429
Greenlanders, form of their skulls . xviii 378
GREENSMITH, case of, tried for murder x 144
GREGORY, Dr. G., his Practice of Medicine . . . . ix 461, 494
on smallpox . . . . . xii 95
vaccination and smallpox . xiv 50
GREGORY, Dr. James, his Conspectus, translation of . . i 520
mode of ventilation by . . xviii 501
on night study . . . i 342
eulogy on . . . . . i 36
GREGORY, Dr. John, anecdote of . xvii 131
GREGORY, Dr. W., Turner's Chemistry xxiii 584
Greifswald, clinical institution at . ii 176
medical school of . . . i 299

Greifswald, university of, account of . i 294

Greiner, Dr., on rheumatic diseases xiv 412, 421

outburst of mysticism from . xiv 415

Grew, his discoveries . . . xiv 484

Grief, benevolence a remedy for . xii 152

Griffin, Dr., on abdominal inflammation xii 280

on laryngismus stridulus . v 595

spinal irritation . . iv 252

Griffin, Messrs., their medical problems xxi 92

Griffith, Dr. J. W., his Chemistry of Blood, &c. . . . xxiii 254

his Manual on urine, &c. . xviii 228

Griffith, Dr. R. E., his introductory lecture . . . iv 193

Griffith, Mr., on hydrocephalus . i 535

Griffiths, Mr., his Chemistry of the Four Seasons . . xxiii 238

*Grinders*, and guide-books . . vi 202

Grinders, health of different kinds of xviii 308

magnetic mouth-piece for . xviii 307

of forks, mortality-table of . xviii 309

unhealthiness of . . xviii 308

pulmonary affections of . . xviii 303

Grinder's asthma, morbid anatomy of xiv 580

objections to the term . xviii 304

dust-protector, Dr. Holland's . xviii 307

rot or asthma, Dr. Favell on . xxii 320

Grinding of tools at Sheffield . xviii 303

Griscom, Dr., on animal mechanism ix 537

Grisenthwaite, Mr., on food . vii 538

Grisolle, Dr., on cecal tumours . ix 445, 458

on lead colic . . . . v 55

on pneumonia . . . xiii 382

Grohmann, Dr., on plague-contagion xix 41

Groin, anatomy of the . . viii 542, xx 139

Gröningen, university of . . vi 277

Gross, Dr., his pathological anatomy xxiii 64

on wounds of the intestines . xxiii 1

Grosse, Dr., his homœopathic practice xxii 568

Grove, Mr., on physical forces . xxiii 247

Growth, fever of, Prof. Reich on the ii 219

of parts, observations on . xxii 267

Growths, development of . . xxi 422

in urethra, instrument for . iii 395

morbid, Mr. Liston on . . xxii 374

origin of . . . i 120

Gruber, Dr., on puerperal fever . vi 539

Gruithuisen, on solution of the stone . . . xii 399, 405

Gruner's Homœopathic Pharmacopœia xxii 570

Guaiacum, its use in syphilis . . v 20

Guano as a manure, Liebig on . xvii 226

supplies ammonia to plants . xi 441

Guards, diseases of lungs in the . viii 225

Gubernaculum testis, structure of . xxii 202

Guerin, M., his unfair claims of merit . . . . xiv 136

on bony deformities . . xii 177, 186

section of muscles . . xi 250

subcutaneous operations . xiv 136

Guggenbuhl, Dr., his cretin institution xvii 515

on cretinism . . . xvii 514

tribute of praise to . . xvii 516

Guiana, British, Dr. Hancock on . i 508

equable climate in . . i 508

for cases of phthisis . i 508

Guibourt on leeches in France . i 281

Guide-books and 'Grinders' . . vi 202

Guide-books, military medical . xix 377, 380

Guide of the practical physician . xviii 285

Guides of medical practice, demand for xviii 285

Guilt, moral, boundary of . . xv 422

Guinea-worm, effects of breaking . xii 429

origin and progress of . xii 429

symptoms from the . xii 429

treatment of . . . xii 429

Guislain, Dr., on gangrene of the lungs . . . . ii 546

on tonics in insanity . . iii 223

Gullet, muscular fibre of the . . xiv 571

Gulliver, Mr., his translation of Gerber . . . xiv 478, 479

on absorption of bone . . vii 140

blood-corpuscles, xi 448, xiv 571, xxi 544

cysticercus . . . xiv 49

diseased arteries . . xviii 366

fibrin . . . . xv 234

pus-like globules . . xv 234

softening of fibrin . . ix 436

suppuration . . . vi 553

the works of Hewson by . xxiii 251

Gully, Dr., his translation of Magendie's formulary . ii 207

on neuropathy . . . iv 471

on simple treatment of disease xv 405

physiological propositions of . iv 473

Gulstonian lectures of Dr. Philip, 1835 ii 129

for 1847 . . . xiv 197

Gum, as an aliment, remarks on . xvii 13

origin of tissues of plants from ix 497

the pabulum of plants . . iv 26

starch, and sugar, similar . xiv 304

Gums, cancer of . . . . xxii 294

coloration of, by lead . . x 427

effect of lead on the . . xi 450

infants', daily scarification of . xxiii 197

state of, in scurvy . . . xx 151

Gun-firing, direction of, detected . v 179

Gunjah, or Indian hemp, use of . x 225

Gunshot wound of knee-joint . xii 330

wounds, of bones . . . xv 401

of head, Mr. Guthrie on . xv 178

on the aperture in . . x 53

table of . . . . xxiii 79

tracing the ball in . . vi 159

treatment of . . . xv 401

use of cold water in . iii 120

Gunther on nature and art curing diseases . . . . i 191

Günzburg, his pathological histology xxiv 118

on percussion, &c. . . xxii 232

Gupta, his edition of the Susruta . xxiii 521

Gurjun balsam, account of . . xvi 356

Gurlt, Dr., his physiology . . vi 513

on structure of the skin . . ii 429

Gusserow on medico-legal chemistry v 212

GÜTERBÖCK, Dr., on Schonlein's discourses . . . . xxi 20

GUTHRIE, Mr. on injuries of the head xv 161, 179

on prevention of disease . . xx 202

on the urinary organs . . xvii 205

GUTHRIE, Mr. Charles, on cataract xx 512

Gutta serena, case of . . . iii 519

Gutting school, reviewers of the . xvii 119

GUY, Dr., his Hooper's Vademecum xiv 217

on duration of life of peers . xxi 1, 9

forensic medicine . . . xvii 228

lung-tests . . . xiii 572

medical education . . xxiii 242

the effect of employments on health . . . xx 421

the health of towns . . xxiii 240

the pulse . . . . vii 458

in phthisis . . ix 337

GUYOT, M., on fracture of the femur iii 238

on heat in ulcers, &c. . . xix 182

on wounds . . . ii 263

Guy's hospital, account of . . i 543

Hospital Reports i 543, ii 198, iii 142, iv 349, vi 60, vii 443, ix 370, xii 326, xiv 122, xvi 308, xviii 332, xxiii 416

engravings of . . iii 142

lying-in charity, reports of . xii 343

Gymnasia of the Greeks . . xvii 458

Gymnastic adjuvants for females . v 407

exercises, remarks on . . v 384

training, use of . . . v 142

Gymnastics, effect of, on health . xx 109

mental, course of . . . xxi 443

Gymnotus, electrical lobe of . . xxii 498

Gynoplastic operation . . . xxi 327

Habit, influence of . . . v 518
of body, elements of . . xxiv 304
the full, bloated, depletion in . iv 188
Habits, influence of, on health . xix 56
medical, in chronic diseases . xxii 454
of body, morbid . . . xxiii 59
Habitual or skilled actions . . xxiv 193
Hachish, Dr. Moreau's studies on . xxiii 217
effects of, in the bodily system xxiii 220
mode of preparing . . xxiii 217
psychical effects of . . xxiii 223
HADFIELD, criminal case of . . xv 88, 93
HADWEN, Mr., on popliteal aneurism vii 153
HAESER, Dr. his researches . . xi 515
HAGSTRÖM on nux vomica in dysentery i 256
HAHNEMANN, a nostrum sold by . xxii 565
life and character of . . xxi 226
on homœopathy . . . xxi 225
on itch and chronic diseases . xxi 232
Hakims, Indian, jealousy of . . xix 77
Hair, ball of, in abdominal abscess xxii 375
characters in races from . xxiv 71
coloration of the . . . ii 451
cortical part of, structure of . xiv 271
development of the . . xiii 525
-follicles, and sebaceous glands ii 453-5
Hair, growing from mucous membrane xxi 204
in posterior chamber of the eye viii 256
invisible or downy, of the body xxi 203
knob of the, structure of . xiv 272
medullary portion of, structure of xiv 271
minute structure of . . xiv 271
sudden change of colour on . xxi 203
structure of, report on . . xix 569
and development of . xxi 202
Wendt on formation of . . ii 451
-worm, common in Dublin . v 595
youthful-like, in old age . vi 80
Hairs growing on the tongue . . xv 235
Hairy hearts, stories of, explained . iv 42
HALES, Dr., his discoveries . . xiv 485
Half-crowns, case of seven swallowed iv 251
HALFORD, Sir Henry, obituary of . xix 315
HALL, Dr. Marshall, an objection to his system . . . xvii 177
doctrine of, anticipated . . v 623
exposition of his reflex theory xvi 159
his case of anasarca . . ix 276
claims stated . . . ix 580
classification of nerves . vi 217
Gulstonian lectures . . xiv 197
introductory lecture . . i 203-4
Io pæans xxiii 193, 195, 197, 198
lamentable conduct . . xxiii 216
letter to his reviewer . iii 577
Observations in Medicine . xxiii 185
practical observations . xix 561
reprints and book-making xxiii 199-201
reclamations answered . xii 201
statement of his claims . xvii 171
views anticipated by Prochaska and others . xxiii 202
wrath at Dr. Forbes . . xxiii 203
melancholy exhibition of . xii 208
mistake respecting . . xiv 524
note on a mistake of . . v 539
on diagnosis, notice of . . i 204
his alcoholic lotion . . xxiii 187
his claims of discovery . v 537-9
his own merits . . . xxiii 195
irritability in paralysis . ix 438
neuralgia criticised . . xiii 158
pathology of the nerves . xi 452
reflex function, &c. . . iii 33, 577
respiratory motions . . xiii 223
the nervous system iii 1, v 486, xii 200, xiv 55, xvi 158
the true spinal marrow . xvii 171
the vis nervosa of Haller . xi 522
plan of observation by . . xiv 55
remarks on his discoveries . iii 580
style of his lectures . . i 205
tribute to . . . . . viii 511
unworthy fretfulness of . . xii 207
value of his discoveries . . xi 453
HALL, Dr. J. C., on diseases of the eye xvi 270
HALL, Mr., on strabismus . . xi 474, 492
HALL, Rev. Robert, his account of his treatment . . . xi 105

Hall, mineral waters at . . xiv 357
Hallé, epistle to, and account of . xix 124
trait in the character of . . xxiii 468
Halle university, account of . . i 294
Haller, his doctrine of irritability iii 3
his doctrines of physiology . v 78
his notions of the nervous system ix 110
his physiology, Dr. W. Clark on i 386
Haller's acid elixir . . . vi 329
Halliday, Sir Andrew, obituary of ix 296
on health of troops . . viii 211
Hallmann, M., on the temporal bone vi 201
Hallucination in cerebral disease . xv 421
Hallucinations and illusions . . xx 22
definition of . . . . x 166
Halves of the body, development in xii 76
Hamilton, Dr. G., on physiology . ix 535
Hamilton, Dr. J., his character as
a teacher . . . . . i 37
his death . . . . . i 308
his letter to the editors . . iv 181
obituary of . . . . . ix 292
on midwifery . iii 131, iv 181, 398
Hamilton, Mr., on wounds of nerves vi 271
Hammick, Dr., harsh style and errors of vi 505
his Mitscherlich . . . vi 505
Hamstring muscles, division of . viii 400
tendons, division of . . ix 229
section of . . . . viii 596
Hamstrings, division of the . . xiii 14
Hancock, Dr., on the climate of Guiana i 508
Hancock, Mr., his translation of Velpeau vi 193
Hand, contractions of, on the cure of xiii 26
gangrene of, case of . . vi 66
section of the muscles of . xiii 26
Sir C. Bell on, remark on . xxi 116
Handloom weavers, health of . xv 299
Handyside, Dr., his case of suicide
vi 455, 473, 476
Hanged corpses, no legal autopsy of
in France . . . . . ii 421
Hanging, case of . . . . . ix 264
cause of death by . . . ii 418
coats of carotids cut by . . ii 421
Dr. Casper on . . . . v 615
during life, new signs of . viii 572
sure signs of . . . . ii 421
emissio seminis in . . ii 421, v 617
erection of penis in . . v 617
experiments on . . . . v 616
marks and ecchymosis from . v 615
medical jurisprudence of . ix 50
non-congested brain in . . xxii 412
Prof. Devergie on death by . ii 418
remarkable case of . . . v 615
semen no proof of . . . . ix 266
signs of . . . . . xii 270
death from . . . ix 266
statistical remarks on . . v 616
strange case of . . . . xii 560
Hannay, Dr., on gonorrhea in women iv 258
Hannover, Dr., his pathology of cancer xxiv 373
on contagious conferva . . ix 550
Hannover, Dr., on exhalation of
carbonic acid . . . xxii 189
on nervous system xvii 379, xviii 134, 140
Hanoverian annals of medicine . iii 460
Hanwell asylum . . . . . ix 145
and Dr. Conolly . . xi 515
bazaar in . . . ix 185
deaths and cures in . xi 128
construction of . . ix 180
cures in . . . . vii 50
devices in, for the insane . xix 598
Dr. Conolly's report on xi 104, 126,
xv 218
Dr. Marx's account of . xv 25
effects of non-restraint at xv 219
entertainments of insane at xv 221
instruction of insane at . xv 222
Justices' report on . . xiii 286
library in . . . ix 185
management of . . ix 182-6
matron of . . . xi 128
medical pupils in . . xiv 218
public worship at . . xii 56
report of . . vii 54, xiii 213
on coercion of lunatics ix 161
royal fund for . . xi 271
seclusion at . . . xi 546
shocking scene in, formerly xxiv 159
treatment of the insane at xiii 274
well-merited motto for . xv 223
Happiness, aggregate and individual i 322
connected with longevity . i 319
Dr. Southwood Smith on . i 321
moral, from hachish . . xxiii 221
principle of the greatest . i 318
the fountains of, in man . i 321
Hardening children, remarks on . ii 126
Hard, calcareous water, calculus from xi 342
palate, on the nerves of the . xiv 222
Hardwiger, on the ophthalmic
clinic of Vienna . . . x 1
Hare, Mr., on spinal curvature . ix 244
Harelip, Dieffenbach on operating for xxi 303
operation for . . . xiv 234
history of . . . xvi 50
in an infant . . . xiv 250
Harem, medical visit to a Turkish . iv 386
Harlan, Dr., his medical researches ii 518
on expiration . . . . ii 518
on the brain of man and animals ii 518
Harmattan, the, its effects on epidemics i 348
Harmony of numbers, remarks on . xviii 170
Harpocrates, the Egyptian demigod xix 121
Harrison, Dr. Edward, his pathology vi 289
obituary of . . . . . vi 289
Harrison, Dr. Edwin, on pectoriloquy iii 554
Harrison, Dr. J., on the nervous
system . . . . . xviii 232
Harrison, Dr. R., on tubercles . iv 244
Harrison, Mr., confused writing of xvi 342
on spinal deformity . . xvi 341
plagiarism of . . . . xvi 344
Harrowgate salts, artificial . . xiii 575

Harrowgate water, artificial . . xiii 575
Harthausen, M., on syphilis in horses xii 494
Harty, Dr., on dysentery . . xxiv 333
Harvest work by teetotallers . . xxiv 536
Harvey, remarks on . . . . xv 449-50
delightful eulogy on his mother xxiii 249
his discoveries disputed . . x 238
his first thought of the circulation v 88
his views of life of the blood . i 388
Mr. Whewell on his discoveries v 88
records of, by Mr. Paget . xxiii 249
Haslar, naval lunatics at, treatment of xvii 285
Hassall, Mr., his microscopic anatomy . . xxii 552, xxiii 252
Hasse, Dr., his morbid anatomy . xv 83-4
on diseases of the respiratory organs . . . . . xxiii 251
Hassing, M., on iodine in syphilis xx 482
Hastings, Dr. C., on natural history iv 197
Dr. Barlow on portrait of . viii 304
some account of . . . iv 559
speech of . . . . . ii 605
Hastings, Dr. John, cases of phthisis cured by . . . . . xvi 496
grounds of disbelief of . . xvi 498
his marvellous cures . . xvi 495
on naphtha . . . . . xvi 490
self-deception of . . . xvi 499
special enormity of his book . xvi 491
specimens of his style . . xvi 492
Hauck, Dr. on syphilitic diseases . x 382
Havard (U.S.), medical school of . ii 586
Haverfordwest lunatic asylum . xix 323
Haversian canals, of the bones . xiv 269
osseous laminæ in . xxiv 394
gland, congenital luxation from hypertrophy of . . xxi 398
Hawkins, Mr. C., on cancer of thyroid gland . . . . . xx 85
on diseases of the face . . vii 143
foreign bodies in larynx . xi 452
spinal cancer . . . xiv 53
tumour of the neck . . ix 440
warty cicatrices . . ii 161
Hawley, Dr., on self-motion of blood iii 258
Hay asthma, case of . . . . xx 245
fever, or summer bronchitis . xx 450
Hays, Dr., his American Cyclopædia i 152
Hayward, Dr., his physiology vii 214, 216
Headache, acid remedies for . . xix 29
after labour, treatment of . ii 86
classification of . . . . xii 104
cured by leeches in the nose . iv 251
ol. terebinthinæ . . xii 432
Dr. Hull on . . . . . xvii 202
from undue lactation . . xii 329
in hypertrophy of heart . . xxii 425
intense, from disease of heart . ii 310
leeches to the nostrils for . xvii 203
nervous, aconite in . . . x 584
remarks on . . . . xiii 147
treatment of . . . xvi 362
periodical, Dr. Hunt on . . xviii 65
Headache, plethoric, on cupping in . xvii 202
scarifying nostrils for . . xvii 203
sedative lotion for . . . xi 235
sick, from constipation . . x 42
use of aconite in . . . xx 467
aconitum napellus in . v 275
Headaches, sick, frequent cause of . iv 62
Head, Sir F., his Bubbles from Brunnen xiv 317
Head and face, case of tumours of . xv 153
malignant diseases of . xiv 580
and liver, sympathy between . ii 10, 11
and neck, Mr. Cock's anatomy of i 541
Head, artificial flattening of the . x 480, 483
blood in, experiments on . xxii 408
in fever . . . . iii 324
blow on, occasional benefit of viii 557
cool, feet warm, controverted . xvii 155
case of impacted . . . ix 262
injury of . . . viii 577
caustic potash to crown of . vii 65
death from blows on . . v 173
determination of blood to xvii 200, 487
diminution of blood in . . iii 324
effused blood in, from violence v 173
diseases of, in India . . xxiii 119
fatal wound of . . . vii 259
flattening of, Indian process of xviii 224
fœtal, Dr. Naegele on full-grown xix 61
impetus of blood in . . iii 324
injuries of . . . . . xiii 123
diagnosis of . . . xiii 130
ecchymosis of eye in . xii 550
extravasation in . . xv 172
insidious cases of . . xiii 129
John Hunter on . . v 52
mania after . . . xv 168
Mr. Guthrie on . xv 161, 179
reckless bleeding in . xvii 200
injury of, abscess of liver after ii 10
instructive case of . . xiii 129
interesting case of injury of . vii 216
keeping it warm . . . xvii 155
Mr. Guthrie on gunshot wounds of xv 178
obstruction to blood in . . iii 324
peculiar circulation in . . iii 323
position of, experiments on . vi 144
presentation of, in labour xii 464, xix 66
-presentations, classification of xii 465
different kinds of . . xii 466
remarks on . . . ii 75
rheumatism of . . . vi 354
size of . . . . . . xvii 265
wounds of, Chelius on . . xx 115
on insanity from . . xxi 464
Healing, and formative effort, the same xxi 528
art, ancient physicians on the xxiii 38
imperfection of . . iii 200
by adhesion . . . . xviii 280
by granulation . . . xviii 280
by the first intention . . xviii 280
by the second intention . . xviii 280
of a wound, Mr. T. W. Jones on xviii 279
or incarnation of lesions . xxiii 57

